# Tumor‐adjacent tissue co‐expression profile analysis reveals pro‐oncogenic ribosomal gene signature for prognosis of resectable hepatocellular carcinoma

**DOI:** 10.1002/1878-0261.12153

**Published:** 2017-12-12

**Authors:** Oleg V. Grinchuk, Surya P. Yenamandra, Ramakrishnan Iyer, Malay Singh, Hwee Kuan Lee, Kiat Hon Lim, Pierce Kah‐Hoe Chow, Vladamir A. Kuznetsov

**Affiliations:** ^1^ Bioinformatics Institute Singapore; ^2^ Department of Computer Science School of Computing National University of Singapore Singapore; ^3^ Division of Surgical Oncology National Cancer Centre Singapore Singapore; ^4^ Office of Clinical Sciences Duke‐NUS Graduate Medical School Singapore Singapore; ^5^ Department of HPB and Transplantation Surgery Singapore General Hospital Singapore

**Keywords:** adjacent non‐malignant tissue, co‐transcription, hepatocellular carcinoma, personalized prognostic biomarkers, primary tumor, ribosome gene

## Abstract

Currently, molecular markers are not used when determining the prognosis and treatment strategy for patients with hepatocellular carcinoma (HCC). In the present study, we proposed that the identification of common pro‐oncogenic pathways in primary tumors (PT) and adjacent non‐malignant tissues (AT) typically used to predict HCC patient risks may result in HCC biomarker discovery. We examined the genome‐wide mRNA expression profiles of paired PT and AT samples from 321 HCC patients. The workflow integrated differentially expressed gene selection, gene ontology enrichment, computational classification, survival predictions, image analysis and experimental validation methods. We developed a 24‐ribosomal gene‐based HCC classifier (RGC), which is prognostically significant in both PT and AT. The RGC gene overexpression in PT was associated with a poor prognosis in the training (hazard ratio = 8.2, *P *= 9.4 × 10^−6^) and cross‐cohort validation (hazard ratio = 2.63, *P *= 0.004) datasets. The multivariate survival analysis demonstrated the significant and independent prognostic value of the RGC. The RGC displayed a significant prognostic value in AT of the training (hazard ratio = 5.0, *P *= 0.03) and cross‐validation (hazard ratio = 1.9, *P *= 0.03) HCC groups, confirming the accuracy and robustness of the RGC. Our experimental and bioinformatics analyses suggested a key role for c‐MYC in the pro‐oncogenic pattern of ribosomal biogenesis co‐regulation in PT and AT. Microarray, quantitative RT‐PCR and quantitative immunohistochemical studies of the PT showed that *DKK1* in PT is the perspective biomarker for poor HCC outcomes. The common co‐transcriptional pattern of ribosome biogenesis genes in PT and AT from HCC patients suggests a new scalable prognostic system, as supported by the model of tumor‐like metabolic redirection/assimilation in non‐malignant AT. The RGC, comprising 24 ribosomal genes, is introduced as a robust and reproducible prognostic model for stratifying HCC patient risks. The adjacent non‐malignant liver tissue alone, or in combination with HCC tissue biopsy, could be an important target for developing predictive and monitoring strategies, as well as evidence‐based therapeutic interventions, that aim to reduce the risk of post‐surgery relapse in HCC patients.

**Abbreviations**
1‐D DDg: one‐dimensional data‐driven grouping
AFP: α fetal protein
AT: adjacent non‐malignant tissue
BCLC classification: Barcelona clinic liver cancer classification
CI: confidence interval
CPG: common prognostic gene(s)
DEG: differentially expressed gene(s)
FA/GO: Functional Annotation and Gene Ontology
HBV: hepatitis B virus
HCV: hepatitis C virus
HCC: hepatocellular carcinoma
IHC: immunohistochemistry
LCI: Liver Cancer Institute
OS: overall survival
PT: primary tumor
RGC: ribosomal gene classifier
SVR: support vector regression
SWVg: statistically weighted voting grouping
TER: translation elongation and ribosomal
TNM: tumor, node and metastasis

## 1 Introduction

Hepatocellular carcinoma (HCC) ranks fifth among solid tumors and causes 70 000 annual deaths worldwide. It is the third leading cause of cancer‐related mortality in males and is most prevalent in Asia and Africa. Unlike many solid tumors, the incidence and mortality of HCC have increased over the past decade (Ashtari *et al*., 2015). The absence or substantial progress of effective HCC therapies is indicated by the mortality rate, which is equivalent to the incidence rate in most countries (Bruix and Sherman, 2011).

Notable disease treatment challenges include the high heterogeneity of the primary tumor (PT) and the pathophysiological status of the adjacent non‐malignant tissue (AT), which affects 70% of patients after resection or local ablation (Llovet *et al*., 2005).

Recent studies of various cancers (including HCC) have incorporated global molecular profiling using various ‘omics’ platforms (Wang *et al*., 2015a). These studies have enabled the development of multiple multigene prognostic biomarkers for stratifying cancer patients into risk subgroups that are relevant for potential treatments (Hoshida *et al*., 2012). Compared to several other cancers (e.g. breast, prostate and hematological), the molecular markers are not used for the diagnosis or determination of prognosis and treatment for HCC patients. Thus, evidence‐based molecular markers that could accurately and reproducibly predict survival time and response to treatment must be identified (Bruix *et al*., 2016).

However, in this respect, significant challenges might reflect certain caveats: (a) poor clinical reproducibility (e.g. when a biomarker fails in an independent cohort validation) and/or poor genetic reproducibility (e.g. different enriched gene sets/deregulated pathways in an independent validation cohort), which limits or confounds the clinical and therapeutic utility of the biomarker, and (b) high diversity in the genetic status and high technical (e.g. different ‘omics’ platforms) and/or clinicopathological cross‐cohort variability from independent clinical centers. To overcome these obstacles, a workflow for biomarker selection that identifies the most etiologically and pathobiologically essential genes, gene products and biological processes with high reproducibility and prognostically confident molecular patterns is needed.

It has been reported that AT substantially contributes to HCC and has an independent prognostic value (Hoshida *et al*., 2008), presumably reflecting the *de novo* multicentric occurrence of HCC in cirrhotic tissue, which impacts late HCC recurrence (> 2 years recurrence‐free survival). Alternatively, PT cells that remain after resection can disseminate across the AT of specific HCC patients to contribute to early HCC recurrence (≤ 2 years of recurrence‐free survival) (Hoshida *et al*., 2008). However, the relationships between the PT and AT can be more complex.

A resurging interest in cancer cell and host tissue interactions, including metabolism, biogenesis and secreted metabolites, metabolic reprogramming in the systemic modulating of gene expression, and signaling pathways in joint cancer‐host tissue compartments, has been observed in recent years. According to the ‘field cancerization’ model (Vauthey *et al*., 2000), pathological and genetic changes in tissues peripheral to a tumor could result from ‘preconditioning’ of the affected organ by various carcinogenic agents. For example, most HCC arise in the background of chronic liver disease [e.g. hepatitis B virus (HBV) or hepatitis C virus (HCV) infection, hepatitis and cirrhosis]. After the surgical resection of PT in the absence of effective therapy of the background medical condition(s), the significant probability of the appearance and development of tumor(s) in field cancerization may be similar to those that prompt the primary HCC.

Alternatively, PT cell growth and progression can modulate the host tissue metabolic pathways and induce epigenetic reprogramming in AT (Skill *et al*., 2011). These changes often lead to tumor‐like functional modulations of the gene expression profiles in non‐malignant tissue cells (Lou *et al*., 2009). For example, the DNA methylation status of a tumor suppressor regulatory signal(s) in the AT can not only be distinct from that of the normal (Arai *et al*., 2009) and cirrhotic liver tissues of non‐HCC individuals, but also similar to that of the PT (Lou *et al*., 2009). In addition, multiple metabolic coupling between PT and AT has been described. Experimental models have been developed (Shapot and Potapova, 1986; Shapot *et al*., 1972) of tumor–host tissue metabolic relationships demonstrating the competition of the tumor and host tissues for RNA precursors and other metabolites. These studies also characterized a phenomenon of the tumor capacity to act as ‘a trap’ for nitrogen and glucose, leading to tumor‐like host cell metabolism and host tissue metabolic ‘assimilation’ peculiarities in glycolysis and RNA biosynthesis.

Cell–cell interactions, external regulatory signaling factors and the active transport of biomolecules that are secreted from PT via the exosomes/microvesicles may induce and maintain the specific tumor‐driven pro‐oncogenic biological processes and signaling pathways relevant to RNA biosynthesis and the ribosomal biogenesis in AT. We propose the presence of multiple tumor‐producing molecular signals, secreting factors and tumor‐induced external metabolite trapping processes that collectively establish tumor‐like common RNA biosynthesis and ribosome biogenesis patterns and specific steady‐state aberrant pathways in AT cells. The interactions between tumor and host tissue compartments assume the co‐microevolution of metabolic processes in AT and AT cell populations during the course of cancer cell metabolism, genetic and epigenetic alterations, cell growth, death and migration, clonal selection and other factors of tumor progression.

In an effort to identify HCC biomarkers, many studies have developed models of gene expression profiling to discover biomarkers either in PT or AT samples (Hoshida *et al*., 2012). Some studies have additionally used the differentially expressed genes (DEGs) between PT and AT as prognostic biomarkers at the prognostic variable pre‐selection step (Mah *et al*., 2014; Orimo *et al*., 2008). By contrast, in the present study, we hypothesize that the next‐generation prognostic biomarkers for HCC prognosis and prediction can be determined via the automatic selection of a subset(s) of co‐expressed survival significant genes in PT and AT pairs. This strategy uses the concepts of (a) field cancerization and (b) PT‐initiated biogenesis assimilation in AT compartments as systemically affected by aggressively growing PT compartments. To our knowledge, such an approach to tumor‐host tissue co‐evolution analysis has not been considered previously in the literature in the context of prognostic/predictive biomarker discovery and disease risk stratification.

We aimed to develop a prognostic biomarker discovery model that would (a) identify common pro‐oncogenic biological processes induced by aggressive PT growth in PT and AT and (b) select variables predicting HCC patient risks after curative resection. We suggested that the expression of potential prognostic genes pre‐selected in the cells of AT, as a non‐malignant cell population, was more genetically stable and less vulnerable to oncogenic reprogramming compared to the pre‐selected genes in the PT cell population. We also assumed that the identification of statistically enriched gene subsets/pathways/biological mechanisms consistent in AT and PT pairs is another important step toward further shortlisting the most biologically/pathologically essential gene candidates for biomarkers. We expected that the use of these two strict pathobiology‐based pre‐selection criteria would eliminate numerous indirectly correlated but non‐essential gene confounders with less or nonreproducible mechanistic functions and prognostic abilities.

In the present study, we specified both a genetic and metabolic preconditioning ‘field cancerization’ model in PT and AT and a PT‐like AT cell ribosomal biogenesis model. We analyzed the role of gene expression patterns in cancer predisposition and metabolism, coupling cancer and non‐malignant host tissues as an interconnected two‐compartmental tissue cell population system.

We developed a hypothesis‐driven HCC biomarker discovery and statistically‐based computationally prediction method. We selected a specific group of common prognostic genes (CPGs) co‐expressed in both PT and AT, generating HCC patient risk stratification into relatively favorable and unfavorable disease outcome subgroups. The CPG analysis enabled the identification of translation elongation and ribosomal (TER) gene subsets with common pro‐oncogenic prognostic patterns as the most over‐represented gene subsets in PT and AT. We showed how the data analysis, integrating paired PT and AT samples, identified the HCC prognostic ribosomal gene classifier (RGC). This prognostic classifier demonstrated high confidence, robustness and reproducibility across the studied cohorts and identified the clinically and pathobiologically reproducible low‐ and high‐risk patient subgroups with thoroughly characterized and druggable deregulated biological pathways. In summary, these results highlight the importance of the deregulation of the TER pathway in HCC, comprising the essential pathological feature common to PT and AT. We discuss the clinical perspectives of these findings and the potential applications of these tools and also propose a novel strategy for the identification of uniformly co‐regulated, pathway‐specific, statistically reliable and reproducible prognostic biomarkers.

## 2 Materials and methods

### 2.1 HCC patients and tissue samples

We retrospectively analyzed the hepatic tissue samples of HCC patients from Singapore, which were collected after surgical treatment with informed consent from the patients. The patients underwent radical resection and follow‐up between 2000 and 2013 at Singapore General Hospital, and their hepatic tissue samples were collected at the National Cancer Centre of Singapore/SingHealth Tissue Repository. HCC diagnosis and treatment for the HCC patients were based on established histological criteria (International Working Party, 1995). After surgical treatment, patients were followed up at least once every 3–6 months. Other post‐surgery patient treatments included imaging and α fetal protein (AFP) monitoring on each follow‐up. The Singapore HCC cohort (Singapore cohort) presented here included only HCC patients diagnosed with resectable HCC, with good liver function (Child status A or B), adequate future liver remnant and good general health. The HCC patients with unresectable lesions with poor liver function or general health (Chow *et al*., 2016) were excluded from the analysis. Linked clinical and histopathology data collected from the patient medical records were rendered anonymous. The study was approved by the SingHealth Institutional Review Board. Cost and practical issues restricted the primary sample size in the present study to 125 HCC patients. PT and AT liver samples obtained at the time of definitive surgery were snap‐frozen and preserved at –80 °C. The inclusion of matching AT samples in the study was based only on the availability of the samples from the SingHealth Tissue Repository and satisfactory results of RNA and microarray quality control. A paired sample design was used and clinical data were available for analysis only after re‐identification of the RNA samples and full completion of microarray profiling.

Median follow‐up in the Singapore cohort was 1.17 years. The detailed patient and tumor features of the Singapore and the Liver Cancer Institute (LCI) cohorts are presented in Tables 1 and S1.

**Table 1:** Clinicopathological characteristics in HCC patients’ cohorts used in the present study

	Clinical characteristic	Singapore (training) (*n* = 115)^a^	LCI (validation) (*n* = 206)^a^	*P*‐value
1	HBV infection (yes/no/–/%)	53/43/19/46	187/4/15/91	< 0.001^d^
2	HCV infection (yes/no/–/%)	20/36/59/18	–	–
3	Sex, male (yes/no/%)	93/22/81	183/23/89	0.06^d^
4	Age (years) (≥50/<50/median)	104/11/64	120/86/51	< 0.001^d^
5	AFP (> 300 ng·mL^−1^/≤ 300 ng·mL^−1^/–/%)	42/68/5/37	94/109/3/46	0.12
6	Cirrhosis (yes/no/%)	62/53/54	189/17/92	< 0.001^d^
7	Multinodular/solitary tumors (yes/no/%)	26/89/23	40/166/19	0.5^d^
8	Tumor size (> 5 cm/≤ 5 cm/%)	65/50/57	71/134/34	< 0.001^d^
9	Total nodules (yes/%)^b^			
	1	89/77	–	–
	2–7	16/14	–	–
	> 7	10/9	–	–
10	Microscopic vascular invasion (yes/no/%)	45/70/39	–	–
11	Albumin Child points (yes/%)^c^			
	1 point	74/64	–	–
	2 points	34/30	–	–
	3 points	7/6	–	–
12	Bilirubin Child points (yes/%)^c^			
	1 point	107/93	–	–
	2 points	7/6	–	–
	3 points	0/0	–	–
13	Child‐Pugh status (yes/%)			
	Child‐Pugh A	101/88	–	–
	Child‐Pugh B	13/11	–	–
	Child‐Pugh C	1/1	–	–
14	Milan criteria (beyond/within/%)	75/40/65		
15	Edmondson tumor grade (3, 4/1, 2/–/%)^c^	62/50/3/53	–	–
16	Platelets score (> 100/≤ 100/%)	108/7/94	–	–
17	Metastasis(imaging) (yes/no/–/%)	2/110/3/2	–	–
18	Presence of tumor capsule (yes/no/–/%)	40/66/9/34	–	–
19	Extra‐hepatic invasion(histology) (yes/no/%)	1/114/1	–	–
20	Portal vein invasion (yes/no/–/%)	8/102/5/7	–	–
21	Positive tumor margins (yes/no/%)	12/103/10	–	–
22	BCLC staging (yes/%)			
	0	4/3	17/8	0.1^d^
	A	75/65	133/65	0.8^d^
	B	27/23	19/9	< 0.001^d^
	C	9/8	22/11	0.3^d^
	–	0/0	15/7	–
23	TNM staging (II–IV/I/–/%)	58/56/1/50	105/86/15/51	0.9^d^
24	Median follow‐up (OS), years (25–75th percentile)	1.17 (0.45–3.12)	4.36 (1.36–4.80)	< 0.001^e^
25	Overall death, *n* (%)	25 (22)	80 (39)	0.002^d^

a Number of patients/percentage.

b Total nodules, based on histology report, including satellite nodules.

c Albumin Child points, Child‐Pugh Category score: 1 point = > 35 g·L^−1^; 2 points = 28–35 g·L^−1^; 3 points = < 28 g·L^−1^; Bilirubin Child points, Child‐Pugh Category score: 1 point = < 34.2 μmol·L^−1^; 2 points = 34.2–51.3 μmol·L^−1^; 3 points = > 51.3 μmol·L^−1^; Edmondson tumor grade: 1 = Grade 1; 2 = Grade 2; 3 = Grade 3; 4 = Grade 4. –, missing data.

d Fisher's exact test (two‐sided).

e Mann–Whitney test.

### 2.2 Study design and endpoint

The pressent study complied with the recommendations for reporting prognostic cancer biomarkers according to the REMARK statement (McShane *et al*., 2005) (Table S2) and the guidelines for evaluating prognostic biomarkers (Simon *et al*., 2009). The prognostic biomarker(s) demonstrated certain traits: (a) significant prognostic power in the training and validation sample sets; (b) statistically independent prognostic value in a multivariate analysis that included known clinicopathological predictive variables; and (c) significant predictive power confirmed in an external cohort reported by independent investigators using the same technology. The primary endpoint of the study was the patient overall survival (OS). OS was defined as the time between surgical resection and death of any cause at last follow‐up. Other measures of patient benefits from use of prognostic biomarkers, such as disease‐free or recurrence‐free survival, were not used because they are surrogate to OS and may not always translate to longer OS (Goh *et al*., 2016; Llovet *et al*., 2008a). Additionally, OS proved to be useful in large and successful targeted drug therapy trials of HCC (Llovet *et al*., 2008b).

### 2.3 Patient tissue sample processing, gene expression microarrays and quantitative RT‐PCR analysis methods

All tissue samples were uniformly homogenized using a TissueLyser LT from Qiagen (Germantown, MD, USA) in accordance with the manufacturer's instructions. RNA isolation was performed for all tissue lysates. Sample preparation and hybridization of labeled cRNA to the HumanHT‐12 v4 Expression BeadChip arrays (Illumina, Inc., San Diego, CA, USA) were conducted in accordance with the manufacturer's instructions. Data from 10 patients were excluded as a result of low RNA or microarray preparation quality (Kauffmann *et al*., 2009). Finally, we used 115 PT samples and 52 AT samples matched to 52 corresponding PT. After the completion of RNA and microarray quality control, the included samples were matched to corresponding clinical data and re‐identified before data analysis. The quantitative PCR experiments were conducted using a QuantStudio™ 6 Flex Real‐Time PCR system in accordance with the standard instructions for Power SYBR Green master mix from ABI systems (Applied Biosystems, Foster City, CA, USA). The qRT PCR signals were normalized with standard reference TBP and relative fold change abundances for desired genes were estimated.

The microarray data for the gene expression profiling of the HCC patient samples are publicly available at GEO: GSE76427. As a validation HCC cohort, we used publicly available data for the 206 HCC patients from the Liver Cancer Institute (the LCI HCC cohort, Fudan University, Shanghai, China, GSE14520) (Roessler *et al*., 2012) that passed the same microarray quality assessment (Kauffmann *et al*., 2009). We selected the LCI dataset for the Singapore data analysis validation because (a) the LCI group is a relatively large publicly available gene expression dataset (includes 206 paired PT–AT samples); (b) clinical data including patient survival data have been available for these 206 patients, allowing survival prediction analysis for the PT‐AT paired expression dataset to be carried out; (c) Singapore and LCI datasets include mostly Chinese/Asian patients; and (d) patients in both groups have a predominantly HBV‐driven ethology, which potentially could result in deregulated molecular pathways distinct from other available patients groups with predominantly HCV‐driven HCC.

### 2.4 Immunohistochemical staining of formalin‐fixed paraffin‐embedded samples

DKK1 protein expression was determined using a DKK1 antibody (ab61034; Abcam, Cambridge, MA, USA) in liver FFPE tumor sections from six HCC patients and the RGC risk subgroups (HR_T_ and LR_T_) in accordance with a standard immunohistochemistry (IHC) protocol. Automated hematoxylin and diaminobenzidine staining (IHC DAB1 Leica Bond III) using a Leica Bond III Automated Stainer (Leica Biosystems, Wetzlar, Germany) was performed using the protocol: (a) dewaxing; (b) pretreatment; and (c) IHC staining. The dewaxing step was performed at 72 °C, followed by pretreatment (unmasking) with the Bond epitope retrieval 2 (ER2) solution, with washing steps being performed using absolute alcohol and Bond Wash Solution. IHC staining was performed using the primary antibody and the Bond™ Polymer Refine Detection kit (Leica Biosystems) was used for subsequent detection. After staining, the slides were dehydrated in absolute alcohol, cleaned with xylene and mounted with DEPEX mounting media (WVR International, Radnor, PA, USA).

### 2.5 Statistical and bioinformatics analyses

To select the survival significant genes and to stratify patients into the relatively LR and HR HCC subgroups, we used the one‐dimensional data‐driven grouping (1‐D DDg) method that was previously developed and successfully used for survival prognosis (Chan *et al*., 2012; Grinchuk *et al*., 2015; Motakis *et al*., 2009).

We defined the CPG as the 1‐D DDg‐defined survival significant gene, and the expression value was binarized using a 1‐D DDg cut‐off value, with patients being stratified into low‐risk (LR) or high‐risk (HR) subgroups for identical PT and AT samples (e.g. either exclusively tumor suppressor‐like or exclusively pro‐oncogenic in both tissue types from the same patient).

The statistically weighted voting grouping (SWVg) method is a variable selection and multivariate prediction statistically‐based voting prediction method (Kuznetsov *et al*., 2006) that uses the 1‐D DDg‐derived binary variables (predictors) of the patient risk groups (LR and HR) as an input dataset (Motakis *et al*., 2009). Following 1‐D DDg, the input file is used to obtain the statistical voting stratification of a patient from the grouping information generated using the binary variables. For each patient, the SWVg score is calculated based on an optimized number of statistically weighted votes of the binary variables (Chen *et al*., 2017). The estimated SWVg score cut‐off was determined by maximizing the significance of the patient separation into HR and LR subgroups. For each patient, the SWVg calculates the prognostic score quantifying the risk of disease development, rank orders the patients by their SWVg scores and separates the patients into the LR and HR subgroups according to the estimated cut‐off score.

DEGs were identified using the GenePattern portal (Reich *et al*., 2006) and the significance of the RGC subgroup similarity was estimated using the ‘SubMap’ module in the portal (Hoshida *et al*., 2007). For Functional Annotation and Gene Ontology (FA/GO) and Pathway Maps analyses, we used either david bioinformatics (da Huang *et al*., 2009a) or MetaCore (https://portal.genego.com/).

A support vector regression (SVR) analysis of the immunohistochemical section images from formalin‐fixed paraffin‐embedded PT was performed as described previously (Smola and Vapnik, 1997). Briefly, each of the six representative HCC patients (three from the HR_T_ RGC subgroup and three from the LR_T_ RGC subgroup) had 54 (6 × 9) sub‐images in the dataset. All sub‐images from the LR_T_ and HR_T_ patients were labeled ‘0’ and ‘1’, respectively. This dataset was labeled with RGB covariance matrix‐based features and used to train and test the SVR system. The image‐processing pipeline, covariance‐based feature extraction system and SVR system were implemented using imagej, OpenCV, C++ and r (see Supporting information: Image based analysis of immunohistochemistry slides). Categorical variables were analyzed using a two‐sided Fisher's exact test or the Freeman–Halton extension of Fisher's exact test. The Mann–Whitney *U*‐test was applied for continuous variables (cytelstudio, version 9; Cytel, Inc., Cambridge, MA, USA). Confidence intervals for the proportions of agreement were calculated according to the Wilson efficient‐score method (Newcombe, 1998). Univariate and multivariate analyses were performed using the Cox proportional hazards regression model and the r ‘survival’ package.

### 2.6 ChIP‐seq binding regions analysis in the proximal promoters of the RGC and other gene sets

To support the *in silico* prediction of the regulatory roles of *MYC* in the RGC and other gene sets, we used the publicly available ChIP‐seq data for *MYC* from the HepG2 hepatocellular carcinoma cell line (GEO ID GSM822291; see Supporting information: ChIP‐seq analysis).

## 3 Results

### 3.1 Identification of common prognostic genes in PT and AT samples and their specific biological characteristics

We retrospectively analyzed the clinical data and the hepatic tissue samples from 125 HCC patients (Singapore cohort) collected after surgical treatment. The clinicopathological characteristics of the HCC patients are presented in Tables 1 and S1.

**Figure 1:**
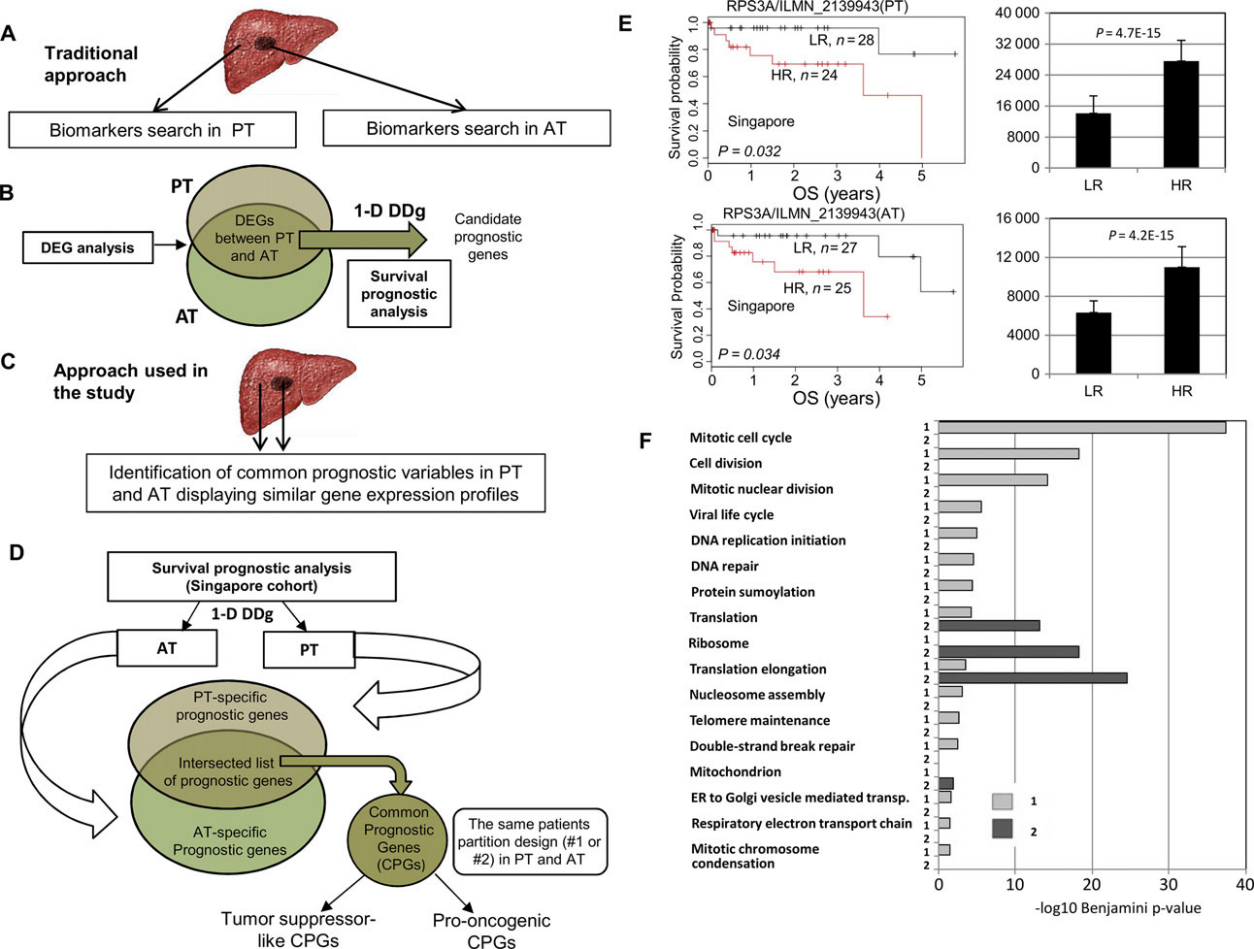
Identification of CPG candidates for PT and AT as HCC prognostic biomarkers. (A, B) Traditional approaches previously used for identifying prognostic biomarkers in HCC. (B) Biomarker candidates may be pre‐selected using DEG analysis between PT and AT, followed by survival prognostic analysis. (C, D) Scheme for identification of tumor suppressor‐like and pro‐oncogenic CPGs in the Singapore HCC cohort using the 1‐D DDg method for survival prognostic analysis. Only the CPG subsets with identical 1‐D DDg design 1 (tumor suppressor‐like CPGs) or only with 1‐D DDg design 2 (pro‐oncogenic CPGs; see 2) in both PT and AT were selected. (E) An example of pro‐oncogenic CPG RPS3A identified in the Singapore HCC cohort. Kaplan–Meier survival curves were obtained using 1‐D DDg by fitting the expression values to survival data. Analyses were performed independently in PT and AT for each gene in the Singapore (*n* = 52) HCC cohort. Vertical bars and *P*‐values show the significant difference in the level of gene expression between the LR and HR patient subgroups (Mann–Whitney test). (F) FA/GO analysis of TER genes in two distinct biological contexts (david bioinformatics software). The results of the FA/GO enrichment analysis are presented for the pro‐oncogenic CPGs subset (1) 1‐D DDg design 2 [the top 1000 survival significant genes obtained according to the scheme shown in (D)] and for the subset of DEGs (2) significantly up‐regulated in PT compared to AT [the top 1000 significantly up‐regulated genes, obtained according the scheme shown in (B)]. Only significant representative FA/GO terms are shown; Fisher test *P*‐values (*P* < 0.05) are Benjamini corrected and –log_10_ transformed.

Figure 1A shows a conventional data analysis schema to identify prognostic biomarkers when a subset of survival significant genes is selected only in either PT or AT to identify diagnostic and prognostic biomarkers. This method may also use DEGs in the analysis and preselection of candidate prognostic genes (Fig. 1B) (Mah *et al*., 2014; Orimo *et al*., 2008). By contrast, the predictor selection and prognostic method identifies the genes (represented by transcript isoforms) exhibiting common expression patterns in both the PT and AT samples termed PT‐AT co‐expressed CPGs (see 2). Here, we describe the procedure for selecting the CPGs used to construct the CPG‐based prognostic classifier to predict HCC patient risks after curative resection.

By definition, the tumor‐suppressor‐like gene has higher expression values in a tissue sample set (e.g. PT or AT) than the corresponding 1D‐DDg‐defined gene expression cut‐off value and the patient belongs to a LR subgroup (1‐D DDg design 1). The pro‐oncogenic gene has higher expression values in a tissue sample set than the 1D‐DDg‐defined gene expression cut‐off value and the patient belongs to the HR subgroup (1‐D DDg design 2).

Figure S1 shows the results of the 1‐D DDg method. We selected fructose‐1,6‐bisphosphatase 1, *FBP1*, encoding the gluconeogenesis regulatory enzyme, acting as a rate‐limiting enzyme in gluconeogenesis and a well‐known tumor suppressor in HCC (Hirata *et al*., 2016; Wang *et al*., 2014), and fibroblast growth factor 1, *FGF1*, encoding a well‐known pro‐oncogenic growth factor in HCC (Lee *et al*., 2015; Yang *et al*., 2017). Figure S1A shows the –log_e_ *P*‐value distribution over the gene expression level domain, and Fig. S1B shows two Kaplan–Maier survival functions representing the relatively LR and HR subgroups derived from 1‐D DDg at the optimized gene expression cut‐off value of *FBP1* indicated in Fig. S1A. As expected, *FBP1* exhibited a tumor suppressor‐like expression pattern. In the case of *FGF1*, a pro‐oncogenic expression pattern was observed (Fig. S1C,D). Notably, the terms ‘pro‐oncogenic’ and ‘tumor suppressor‐like’ are used in the specific context of the survival prognosis analysis (Fig. S1). However, such gene classifications are useful and often correlated with the functional classification of many known tumor suppressors and oncogenes, which are respectively defined based on the pathobiological roles of the genes and gene products in malignant cells (Chen *et al*., 2017).

We designated the CPG as a survival‐significant gene, for which (a) gene expression was binarized according to the 1‐D DDg gene expression cut‐off value; (b) the gene expression cut‐off value was used to stratify the cohort into LR and HR patient subgroups; and (c) the patient survival pattern in the given cohort was identical for PT and AT samples (e.g. either exclusively tumor suppressor‐like or exclusively pro‐oncogenic in both tissue types) (Fig. 1E).

Figure S2 shows the main steps of the workflow, including descriptions of the input and output data sets and the analytical and experimental methods. We used the Singapore cohort to select CPGs observed in the 52 PT‐AT paired samples. We used a multicriteria approach.

In the first step of the CPG selection process, we used 1‐D DDg values as relatively weak selection criteria for the potential prognostic variables (Wald's statistics *P *≤ 0.1), assuming that the next‐level filters for accuracy, robustness and reproducibility criteria will enable optimization of the number and combination of high‐confidence predictors composing the final prognostic signature. The Venn diagram analysis of the 1‐D DDg‐based whole transcriptome profile screen in PT and AT samples resulted in the identification of a large common gene subset (Fig. 1D) comprising 2390 unique (Hg19) gene IDs.

Next, we applied a FA/GO analysis using david, version 6.7 (da Huang *et al*., 2009a) to shortlist the most significantly enriched biological processes and pathways, probably related to the most biologically or pathologically essential CPG subsets (Fig. S2). We identified the gene subsets of the survival significant pro‐oncogenic (e.g. Figs 1E and S3A,B) and tumor suppressor‐like (e.g. Fig. S3C,D) CPGs. We intended to use a sample size‐balanced and multicriteria approach to select unbiased, specific and robust prognostic variables. The 1000 top most significant genes in each CPG subset have been selected. We did this because of a probable effect of sample disbalance: different gene list sizes may lead to a bias in the enrichment analysis test. This sample disbalance also may affect the ranking of large‐sized GO categories, making it difficult to compare gene categories in the gene lists (da Huang *et al*., 2009b).

For the pro‐oncogenic CPG subset, using david, version 6.7, we found that most confidence biological process GO terms are determined by the genes encoding translational elongation proteins (*P *= 2.8 × 10^−25^) and ribosome (*P *= 5.2 × 10^−19^). We also found the moderately enriched term for mitochondrion (*P *= 0.01) (Fig. 1F). Figure 1E shows the results of the survival prediction analysis for the CPG *RPS3A*, the TER gene co‐expressed in PT and AT samples. In each tissue type, *RPS3A* showed a pro‐oncogenic prognostic pattern. The proportion of agreement between the PT and AT stratifications was 0.731 at the 95% confidence interval (CI) (0.587–0.84). Interestingly, no significant FA/GO terms were observed among the top 1000 survival significant tumor suppressor‐like CPGs. Figure S3 shows two examples of the pro‐oncogenic ribosomal CPG *RPL3* and the tumor suppressor‐like *SPOP* CPG according to the 1‐D DDg analysis. Typically, the survival patterns predicted by CPG in PT‐AT pairs were significantly concordant. For example, the proportion of agreement between the PT and AT stratifications given by the 1‐D DDg *SPOP* classifier was 0.673 at the 95% CI (0.528–0.792) and that given by the 1‐D DDg *RPL3* classifier was 0.67 at the 95% CI (0.492–0.767). These findings suggest the pathobiological importance of *SPOP, RPL3* and other translational elongation and ribosome biogenesis CPGs in AT as a novel class of potential non‐malignant tissue clinical biomarkers for malignancy diagnostics, the prognosis of tumor aggressiveness and ‘anti‐cancerization’ tissue therapeutic targeting.

We also analyzed the top 1000 significantly DEGs up‐regulated in PT compared to AT (*t*‐test *q* < 0.05; 115 PT versus 52 AT samples, respectively) (Fig. 1B). By contrast to the pro‐oncogenic CPG subset, the list of the top 1000 DEGs up‐regulated in PT compared to AT, included, as expected, cell cycle, cell division and mitosis gene enrichment; the FA/GO terms essentially predominated over the other FA/GO terms, including TER gene terms (Fig. 1F) (i.e. the FA/GO terms ‘GO:0000278~mitotic cell cycle’ with *P *= 3.7 × 10^−38^ and ‘GO:0006414~translational elongation’ with *P *= 2.9 × 10^−4^). Independently, panther (Thomas *et al*., 2006) identified similar highly enriched and significant FA/GO terms (Fig. S4).

Thus, these findings suggest that, in AT, the genes encoding translation elongation, ribosome machinery components and ribosomal biogenesis in general might pre‐exist in malignant predisposition (e.g. pre‐cancer tissue initiated by mutations, viral, metabolic or epigenetic reprogramming) and/or PT‐activated ‘cancerization’ behaviors. In both cases, pro‐tumorigenic cellular/tissue behavior in AT may be the result of switching‐on/exerting cellular extra‐ribosomal functions (Coulouarn *et al*., 2006; Kim *et al*., 2004; Lindstrom, 2009; Wang *et al*., 2015b). In pathobiological and clinical contexts, the TER CPGs could be considered novel and perspective diagnostic factors of host–cancer interactions and prognostic biomarkers. The prognostic utility of TER CPGs is based on the detection of similar pathobiological alterations and disease outcome prediction patterns in PT and AT. The differences in the expression profiles between PT and AT, as expected, are predominantly defined by cell cycle/mitosis genes. As described below, we performed several basic computational and experimental analyses addressing the pathobiology characteristics of TER CPGs and their clinical significance.

### 3.2 RGC: identification, robustness and reproducibility across cohorts

**Figure 2:**
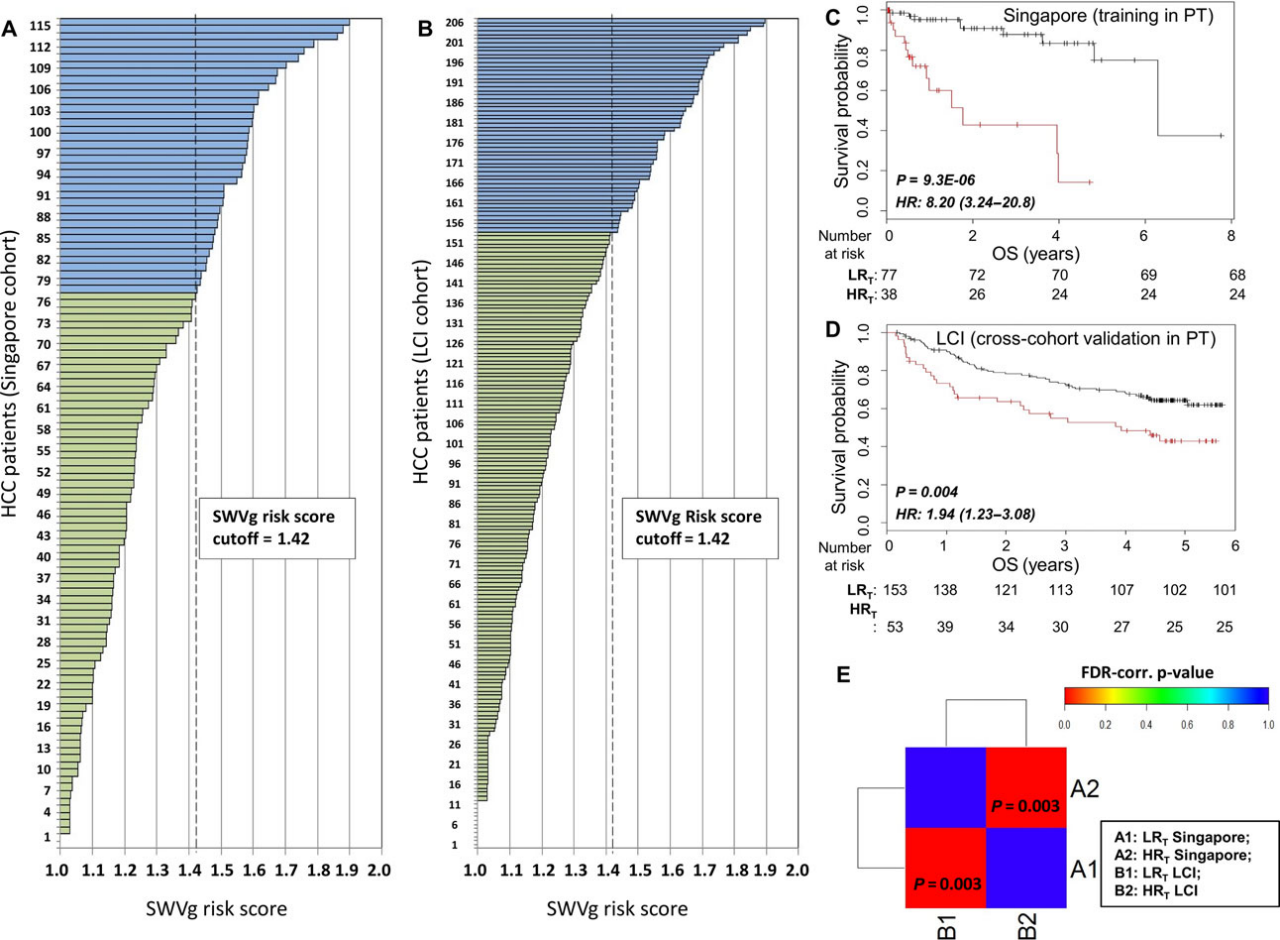
Cross‐cohort validation of the RGC in PT. (A, B) The results of the SWV procedure for the selected 24 ribosomal genes in the Singapore (A) and LCI (B) HCC patient cohorts (see Results). Green: LR HCC patients (LR_T_ subgroup); blue: HR HCC patients (HR_T_ subgroup). (C, D) Kaplan–Meier survival curves for integrated patient partitions in the Singapore and LCI HCC cohorts, respectively. *x*‐axis: OS, years; *y*‐axis: patient survival probability. (E) Subclass association matrix obtained as a result summary of the SubMap analysis (see 3). The bottom left red quadrant indicates the significant similarity between the two LR_T_ subgroups, the top right quadrant indicates the significant similarity between the two HR_T_ subgroups (in the Singapore and LCI cohorts, respectively).

**Figure 3:**
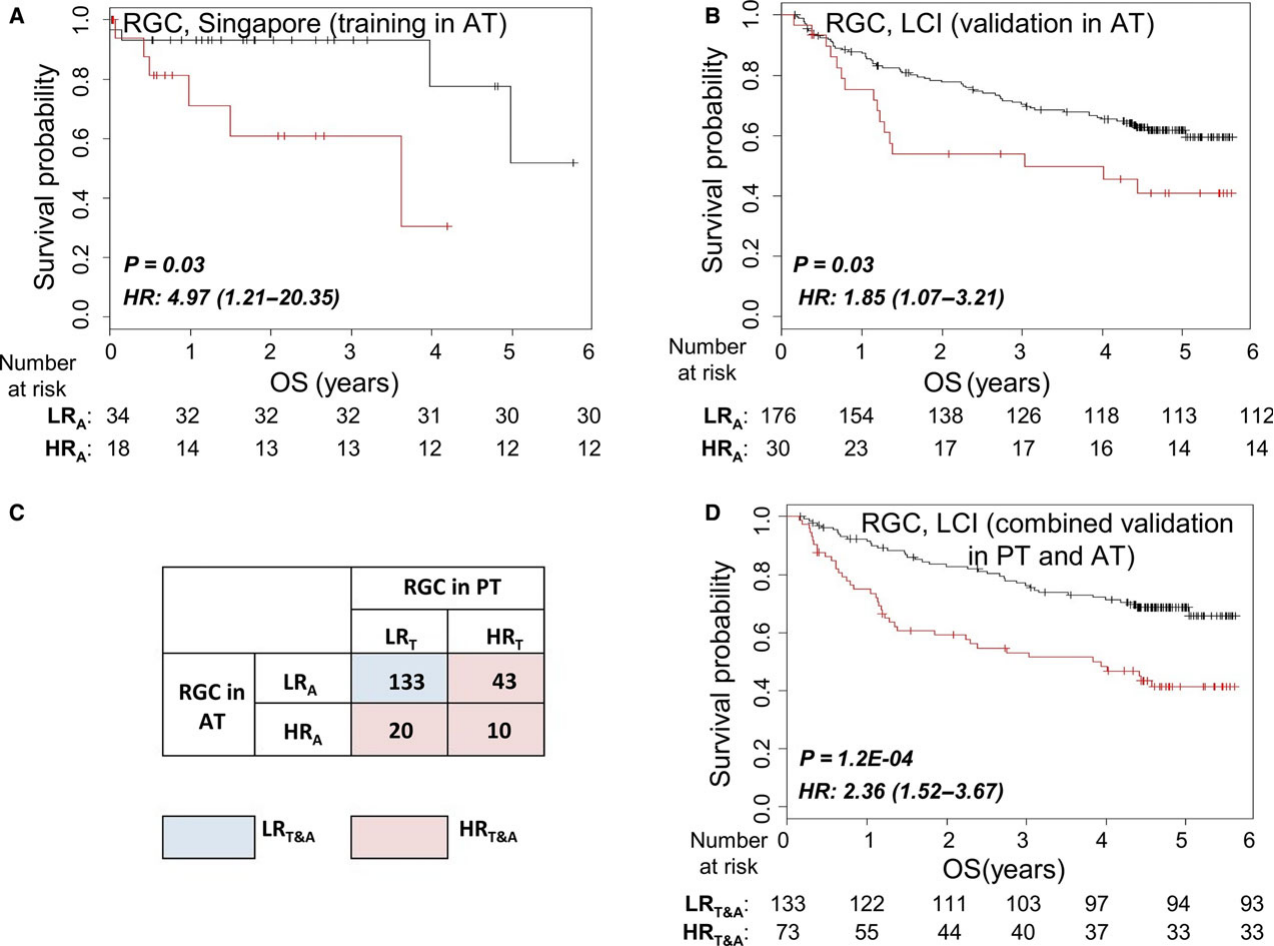
RGC‐based stratification in AT and PT. (A, B) Training and cross‐cohort validation of the RGC in AT. Kaplan–Meier survival curves for integrated patient partitions in the Singapore and LCI HCC cohorts, correspondingly. (C) Contingency table for HCC patient stratification in PT and AT in the LCI cohort. The combined LR subgroup LR_T__&A_ included the patients stratified only as LR using gene expression information from PT and AT (blue). The HR subgroup HR_T__&A_ included the remaining patients (pink). (D) Combined stratification (cohort validation model) using information from PT and AT in the LCI cohort.

Using Singapore cohort gene expression and patient's survival data, we tested the utility of the identified CPGs as multigene prognostic HCC biomarkers. Based on the previous steps of the workflow (Fig. S2), ‘GO:0006412~translational elongation’ displayed the strong term enrichment *P*‐values among the other CPG GO terms (Figs. 1F and S4). We used the 44 gene symbols under this term, referred to as ‘TER genes (Singapore)’ (Table S3). The 44 gene subset includes two translational elongation genes (*EEF1A1* and *EEF1B2*); the other 42 genes were specified by cellular component category as ‘GO:0005840~ribosome’. By using the same method as we applied in Singapore cohort, we identified stratification cut‐off values of individual genes and selected pro‐oncogenic CPGs in the LGI cohort PT – AT paired samples. We also carried out GO enrichment analysis. As result, we identified 60 CPGs (Table S4) specified the highest enriched gene term ‘GO:0006412‐transcriptional elongation’ (Fig. S5A). The next most significant GO term was ‘GO:0005840~ribosome’. Remarkably, an independent identification of the pro‐oncogenic CPGs in the Singapore and LCI cohorts led to a highly significant overlapping between the CPGs identified in the LCI and Singapore cohorts (hypergeometric test, *P *= 1.5 × 10^80^; Fig. S5B). Next, we applied 1‐D DDg to the 115 available PT samples in Singapore cohort as a discovery dataset and selected the CPGs satisfying the Wald statistics cut‐off value at *P* < 0.05. As result of SWVg implementation (see Methods), the 24 most prognostically significant ribosome genes were selected from the PT TER genes (Singapore) and formed our HCC prognostic model (Table S5A). Table 2 provides an annotation of these 24 ribosomal genes. The integrated SWVg risk score was calculated for each individual patient. The SWVg scores for all patients were refitted to the survival data, and the optimal SWVg score cut‐off value for patient stratification was determined (cut‐off = 1.42; Fig. 2A). The classifier successfully stratified the Singapore HCC patients using either PT (Fig. 2C) or AT (Fig. 3A) microarray data sets: *P *= 9.3 × 10^−6^, hazard ratio = 8.20 (3.24–20.8) for PT‐based prognostic stratification and *P *= 0.03, hazard ratio = 4.97 (1.21–20.35) for AT‐based prognostic stratification. We referred to this new CPG‐based HCC classifier as the RGC.

**Table 2:** Pro‐oncogenic ribosomal genes of the 24‐gene HCC prognostic classifier

Number	Host gene symbol	Illumina probe ID	RNA ID	Host gene description (UCSC genome browser)	Chromosome band
1	RPL9	ILMN_1750507	NM_001024921	Ribosomal protein L9	4p13
2	RPL12	ILMN_2116366	NM_000976	Ribosomal protein L12	9q34
3	RPL26	ILMN_1731546	NM_000987	Ribosomal protein L26	17p13
4	RPL37	ILMN_2191634	NM_000997	Ribosomal protein L37	5p13.1
5	RPL31	ILMN_1754195	NM_000993	Ribosomal protein L31	2q11.2
6	RPL41	ILMN_2331890	NM_001035267	Ribosomal protein L41	12q13
7	RPL30	ILMN_1754303	NM_000989	Ribosomal protein L30	8q22
8	RPS9	ILMN_1749447	NM_001013	Ribosomal protein S9	19q13.4
9	RPS15A	ILMN_1787949	NM_001030009	Ribosomal protein S15a	16p12.3
10	RPS25	ILMN_1746516	NM_001028	Ribosomal protein S25	11q23.3
11	RPS11	ILMN_1740587	NM_001015	Ribosomal protein S11	19q13.3
12	RPS4X	ILMN_2166831	NM_001007	Ribosomal protein S4, X‐linked	Xq13.1
13	RPL19	ILMN_1701832	NM_000981	Ribosomal protein L19	17q12
14	RPL32	ILMN_2400143	NM_001007073	Ribosomal protein L32	3q13.3‐q21
15	RPS5	ILMN_1707810	NM_001009	Ribosomal protein S5	19q13.4
16	RPL34	ILMN_1706873	NM_000995	Ribosomal protein L34	4q25
17	RPL3	ILMN_2319994	NM_001033853	Ribosomal protein L3	22q13
18	RPL36	ILMN_1685088	NM_033643	Ribosomal protein L36	19p13.2
19	RPS2	ILMN_2218277	NM_002952	Ribosomal protein S2	16p13.3
20	RPL15	ILMN_1762747	NM_002948	Ribosomal protein L15	3p24.1
21	RPS13	ILMN_1777344	NM_001017	Ribosomal protein S13	11p
22	RPL18A	ILMN_2141452	NM_000980	Ribosomal protein l18a	19p13.11
23	RPS12	ILMN_1782621	NM_001016	Ribosomal protein S12	6q23
24	RPL17	ILMN_1655422	NM_000985	Ribosomal protein L17	18q21

Next, to test the reproducibility and robustness of the RGC, we performed a validation analysis of the classifier using data from an independent HCC cohort (LCI cohort, 206 HCC patients, Methods). In the contexts of robustness and reproducibility of the potential RGC predictors, we carried out a comparison of the 1D DDg results obtained in Singapore and LGI datasets. Our results, presented in Table S5 suggest a high probability of occurrence of the same survival significant genes in PT of LGI cohort and ability to use Singapore PT data as a training set to develop multi‐gene prognostic signature(s) (see also Supporting information: Comparison of 1D DDg results obtained in the Singapore and LGI datasets). The individual stratification gene expression cut‐off values obtained from the 1‐D DDg and the SWVg risk score cut‐off value (cut‐off = 1.42) in the Singapore cohort were used for LCI patient stratification (Fig. 2B). The strict fixation of the pro‐oncogenic type of prognosis variables and parameter values in the 1‐D DDg and SWVg procedures in the Singapore cohort (training) enabled a prognosis prediction blinded to the survival data (defined by overall survival, OS) in the validation (LCI) cohort (at Wald *P = *0.004) (Fig. 2D). The stratification results in two datasets Singapore and LCI (Fig. 2C,D) can be represented by the fractions of patients within each cohort of particular interest, such as HR. This event is represented in the Singapore and LCI cohorts with the proportions 38/115 = 0.33 and 53/206 = 0.26, respectively. We found that the 95% CI for the difference between these proportions was not significant, which suggests subpopulation similarity across different cohorts. Independently, the inter‐cohort subgroups agreement analysis using the SubMap algorithm (Hoshida *et al*., 2007) revealed that the ordered HR_T_ and LR_T_ subgroups based on the results of patient stratification displayed significant agreement between the Singapore and LCI cohorts (Fig. 2E).

Thus, these results suggest that the RGC enables the identification of the robust predicting system for HCC inter‐cohort prognosis.

Finally, we also addressed the question of whether the highly enriched DEGs differentiating PT and AT could provide a reproducible prognostic signature of HCC. Using Singapore data, we selected the DEG subset identified under the three most highly enriched FA/GO terms (‘GO:0000278~mitotic cell cycle’, ‘GO:0051301~cell division’ and ‘GO:0007067~mitotic nuclear division’) (Fig. 1F). These genes were further processed using 1‐D DDg, as described in the Materials and methods. We further selected the genes that displayed significant Wald *P*‐values (*P* < 0.05) in the Kaplan–Meier survival analysis (Table S6). Next, SWVg analysis generated a 41‐gene prognostic signature represented by mitosis/cell cycle genes (Table S6). The 41‐gene prognostic signature displayed significant HCC patient partitioning in the Singapore cohort (Fig. S6A). However, this signature failed in validation in the LCI cohort (using the same procedure as that used for the RGC described above) (Fig. S6B). Non‐significant result we also observed in AT of LCI cohort (Fig. S5D versus Fig. S5C). The lack of reproducibility could be explained by the relatively high inter‐cohort variation in the environmental, ethnic, clinical and pathological parameters that are relevant to cell cycle/mitotic gene expression and relevant pathways.

By contrast, the RGC signature (based on the CPG model; Table S5A) led to reproducible predictions and consistent results for individual predictor genes (see Tables 2 and S5A) and their multigene classifier (Fig. 2C,D; Table S5A).

Altogether, these results indicate the relatively high robustness of the prognostic model to inter‐cohort variations in environmental, ethnic, clinical and pathological parameters to CPGs expression and relevant pathways.

### 3.3 RGC is an independent prognostic factor for HCC progression

The list of clinicopathological parameters available for analysis in Singapore and LCI cohorts is presented in Table 1. For many of these parameters, we detected significant differences in distributions between Singapore and LCI cohorts (Table 1; see also the Supporting information: Comparison of standard clinicopathological parameters between the Singapore and LCI cohorts). Such differences between the cohorts could probably be explained by the differences in the environmental factors, healthcare systems and non‐identical study design between LCI (Roessler *et al*., 2010) and Singapore cohorts and/or various accepted HCC patient diagnoses and treatment guidelines between LCI and Singapore cohorts (Han *et al*., 2011). We used univariate and multivariate Cox regression analyses to compare the prognostic performance of the RGC with these clinical factors (Table 3).

**Table 3:** Univariate and multivariate analyses of clinical, pathological and molecular variables for overall survival in the training, validation and overall cohorts

Univariate analysis					Multivariate analysis				
Variables	Patients (*n*)	Hazard ratio	95% CI	Wald *P*‐value	Variables	Patients (*n*)	Hazard ratio	95% CI	Wald *P*‐value
Training cohort (Singapore) (*n* = 115)									
RGC (R1)	115	8.2	3.23–20.8	9.4 × 10^−6^	RGC (R1)	115	6.4	2.38–17.19	0.0002
Age (> 50 years)	115	1.0	0.96–1.04	0.8	–	–	–	–	–
Sex (female)	115	0.81	0.18–3.54	0.8	–	–	–	–	–
AFP (> 300 ng·mL^−1^)	115	1.49	0.65–3.45	0.3	–	–	–	–	–
HBV status^a^	96	1.74	0.71–4.27	0.23	–	–	–	–	–
Tumor size (> 5 cm)	115	1.33	0.75–3.31	0.5	–	–	–	–	–
Multiple tumors	115	1.71	0.69–4.20	0.24	–	–	–	–	–
Total nodules^b^	115	1.36	0.80–2.30	0.25	–	–	–	–	–
Microvascular invasion	115	1.69	0.74–3.84	0.21	–	–	–	–	–
Albumin child points^b^	115	1.95	1.07–3.57	0.03	Albumin child points^b^	115	2.24	1.07–4.68	0.03
Bilirubin Child points^b^	115	0.68	0.095.05	0.70	–	–	–	–	–
Cirrhosis	115	1.55	0.66–3.66	0.32	–	–	–	–	–
Child status^b^	115	2.02	0.58–6.96	0.26	–	–	–	–	–
Milan criteria^b^	115	0.81	0.29–2.24	0.68	–	–	–	–	–
Edmondson tumor grade^b^	112	0.93	0.52–1.65	0.79	–	–	–	–	–
Presence of tumor capsule	106	1.03	0.41–2.57	0.9	–	–	–	–	–
Portal vein invasion	110	0.90	0.12–6.86	0.9	–	–	–	–	–
Platelet score	115	0.67	0.08–5.08	0.7	–	–	–	–	–
Positive tumor margins	115	3.07	0.98–9.51	0.05	–	–	–	–	–
TNM staging^c^	115	1.72	1.16–2.56	0.007	TNM staging	115	1.21	0.62–2.40	0.66
BCLC staging^d^	115	2.50	1.39–4.49	0.002	BCLC staging	115	1.73	0.66–4.58	0.26
Validation cohort (LCI) (*n* = 206)									
RGC (R1)	206	1.94	1.23–3.08	0.004	RGC	187	1.77	1.02–3.06	0.04
Age (> 50 years)	206	0.99	0.97–1.01	0.32	–	–	–	–	–
Sex (female)	206	0.95	0.55–1.68	0.8	–	–	–	–	–
AFP (> 300 ng·mL^−1^)	205	1.66	1.07–2.60	0.03	AFP (> 300 ng·mL^−1^)	187	1.01	0.58–1–68	0.97
HBV status^a^	191	1.62	0.23–11.68	0.6	–	–	–	–	–
Tumor size (> 5 cm)	205	1.75	1.12–2.72	0.01	Tumor size (> 5 cm)	187	0.81	0.43–1.50	0.5
Multiple tumors	206	1.76	1.08–2.88	0.02	Multiple tumors	187	0.69	0.38–1.26	0.22
Cirrhosis	206	4.35	1.07–17.72	0.04	Cirrhosis	187	3.43	0.82–14.25	0.09
TNM staging^c^	191	1.81	1.44–2.27	4.01 × 10^−7^	TNM staging	187	1.37	0.98–1.92	0.06
BCLC staging^d^	191	2.14	1.65–2.79	1.4 × 10^−8^	BCLC staging	187	1.84	1.23–2.75	0.003
Overall (Training + Validation cohorts) (*n* = 321)									
RGC	321	2.63	1.77–3.92	1.6 × 10^−6^	RGC	302	2.48	1.59–3.85	5.5E–05
Age (> 50 years)	321	1.02	0.64–1.50	0.9	Age (> 60 years)	–	–	–	–
Sex	321	0.66	0.33–1.31	0.24	Sex	–	–	–	–
AFP (> 300 ng·mL^−1^)	318	1.44	1.06–1.96	0.02	AFP (> 300 ng·mL^−1^)	302	0.98	0.71–1.36	0.9
HBV status^a^	287	1.48	0.71–3.08	0.3	HBV status^a^	–	–	–	–
HCV status^a^	56	0.61	0.13–2.90	0.5	HCV status^a^	–	–	–	–
Tumor size (> 5 cm)	320	1.54	1.045–2.28	0.03	Tumor size (> 5 cm)	302	0.93	0.57–1.53	0.8
Multiple tumors	321	1.77	1.15–2.73	0.009	Multiple tumors	302	0.7	0.42–1.17	0.17
Cirrhosis	321	2.08	1.11–3.89	0.02	Cirrhosis	302	1.69	0.85–3.35	0.13
TNM staging^c^	306	1.80	1.47–2.19	6.6 × 10^−9^	TNM staging	302	1.37	1.03–1.82	0.03
BCLC staging^d^	306	2.19	1.72–2.78	2.1 × 10^−10^	BCLC staging	302	1.70	1.20–2.42	0.003

a HBV and HCV status was based on serology and/or documented history.

b Total nodules, based on histology report, including satellite nodules; total nodules are binned as: 1 = 1 nodule; 2 = 2–7 nodules; 3 = 7 or more nodules. Albumin Child points were binned according to Child‐Pugh Category score: 1 point = > 35 g·L^−1^; 2 points = 28–35 g·L^−1^; 3 points = < 28 g·L^−1^; Bilirubin Child points, Child‐Pugh Category score: 1 point = < 34.2 μmol·L^−1^; 2 points = 34.2–51.3 μmol·L^−1^; 3 points = > 51.3 μmol·L^−1^; Edmondson tumor grade binning: 1 = Grade 1; 2 = Grade 2; 3 = Grade 3; 4 = Grade 4; Child status binning: 1 = Child A; 2 = Child B; 3 = Child C; Platelets score binning: 1 = ≤ 100; 2 = > 100; Milan criteria binning: 1 = beyond; 2 = within.

c TNM staging binning: 1 = Stage I (T1, N0, M0); 2 = Stage II (T2, N0, M0); 3 = Stage IIIA (T3a, N0, M0); 4 = Stage IIIb (T3b, N0, M0), Stage IIIc (T4, N0, M0), Stage IVa (Any T, N1, M0), Stage IVb (Any T, Any N, M1).

d BCLC staging binning: 1 = Stage 0; 2 = Stage A; 3 = Stage B; 4 = Stage C. NA, data not available. –, excluded from the analysis. OS was used as the endpoint in all analyses.

In the Singapore cohort, the parameters metastasis and extra‐hepatic invasion were excluded from the analysis as a result of their low informativeness. HCV status was excluded because of a substantial amount of missing data in the Singapore and LCI cohorts (Table 1).

Univariate analysis shows that in addition to albumin expression (*P = *0.03), tumor, node and metastasis (TNM) staging (*P = *0.007) and Barcelona clinic liver cancer (BCLC) staging (*P = *0.002), the RGC classification was a significant indicator for OS (*P = *9.4 × 10^−6^) in the Singapore cohort. In the LCI cohort, significant prognostic powers were observed for the RGC (*P = *0.004), tumor size (*P = *0.01), multiple/solitary tumor formation (*P = *0.02) and cirrhosis status (*P = *0.04). The TNM and BCLC staging displayed the best prognostic performances (*P = *4.0 × 10^−7^ and *P = *1.4 × 10^−8^, respectively). In the univariate analysis, combining the overall data for the training and validation cohorts revealed the following prognostically significant variables: AFP level (*P = *0.02), tumor size (*P = *0.03), multiple/solitary tumors (*P = *0.009), liver cirrhosis status (*P = *0.02), TNM staging (*P = *6.6 × 10^−9^), BCLC staging (*P = *2.1 × 10^−10^) and the RGC (*P = *1.6 × 10^−6^). Interestingly, in additional association analyses of RGC with clinical phenotypes (Table S7), we detected its significant association with AFP levels (*P = *1.8 × 10^−7^) and BCLC classification (*P = *0.004) in the LCI cohort and with AFP level (*P = *1.0 × 10^−3^), multinodular tumors (*P = *0.03) and BCLC staging (*P = *0.005) in the overall cohort (Table S7). However, in the multivariate analysis (Table 3), the RGC classification retained its independent prognostic value (*P = *0.0002 in Singapore cohort, *P = *0.04 in the LCI cohort and *P = *5.5 × 10^−5^ compared to TNM staging (*P = *0.03) and BCLC staging (*P = *0.003), in the overall cohort) (Table 3). These analyses suggest that the RGC could be considered an independent prognostic factor for HCC.

### 3.4 The PT RGC prognostic pattern is reproducible in AT data

We tested the performance of the RGC using the AT expression data of the Singapore (training) and LCI cohorts (validation). For prognosis using AT data, we implemented the same stratification algorithm as implemented for the discovery of RGC in the analysis of PT data (Fig. 2). The use of AT data for RGC training resulted in a significant Singapore HCC patient stratification (the HR_A_ and LR_A_ subgroups, Wald *P* < 0.05) (Fig. 3A). Additionally, the difference between the agreement scores of the HR proportions between PT and AT was not significant, suggesting a similarity of stratification between the PT and AT using RGCs in the Singapore cohort. The strict fixation of the pro‐oncogenic type of prognosis variables and parameter values in the 1‐D DDg and SWVg procedures in the Singapore cohort (training) enabled a prognosis prediction blinded to the survival data in the validation (LCI) cohort (at Wald *P = *0.03) (Fig. 3B).

Notably, the 41‐gene signature based on cell cycle genes generated from DEGs between PT and AT (Table S6) failed in cross‐cohort validation in AT (Fig. S6C,D) and would have questionable clinical utility.

### 3.5 RGC power can be improved by combining PT and AT gene expression data

Next, we proposed that the grouping of common LR patients from relatively low‐risk LR_T_ and LT_A_ prognosis subgroups identified in PT and AT could improve the stratifying power of the RGC. We combined personalized RGC data from the PT and AT datasets and constructed HR_T&A_ and LR_T&A_ subgroups (Fig. 3C). Using LCI data as an example, we observed a positive interaction effect. The Wald *P*‐value in the combined classifier was *P = *1.2 × 10^−4^ (Fig. 3D) compared to *P = *0.003 and *P = *0.03 in the PT‐based (Fig. 2D) and AT‐based (Fig. 3B) stratification prognostic models, respectively. We also tested the other combination scenario, alternative to that shown in Fig. 3C, stratifying common HR patients from HR_A_ and HR_T_ subgroups as a combined HR_T&A_ patient subgroup. However, an imbalanced stratification into risk subgroups (i.e. *n* = 196 in the LR subgroup and *n* = 10 in the HR subgroup) was observed that limits predictive power (not shown data). We concluded that the prognostic power of the original PT‐based RGC can be improved using RGC obtained from AT.

### 3.6 The RGC‐based stratification of HCC patients reveals deregulated pathways in PT and AT

**Figure 4:**
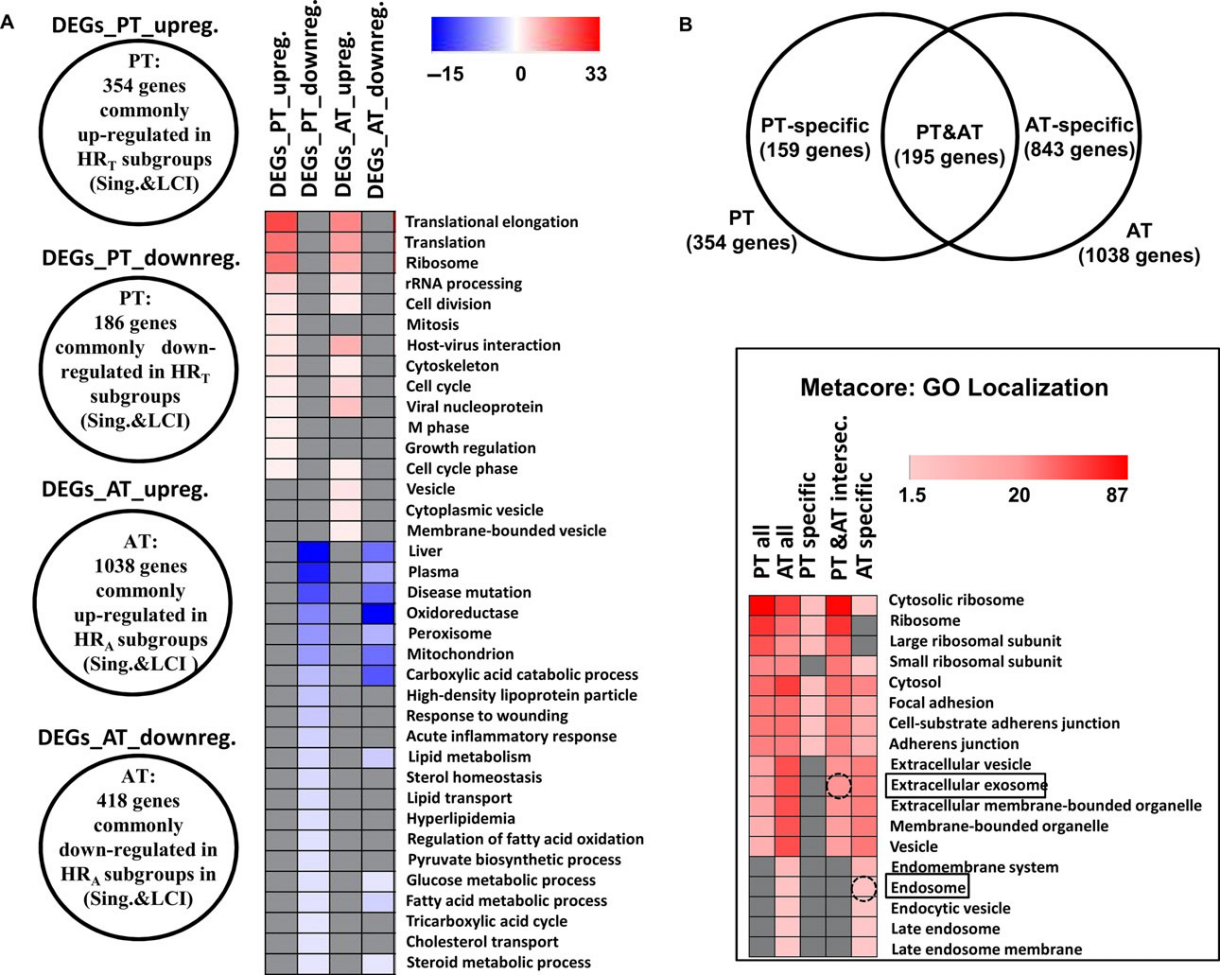
Comparison of biological pathways between the RGC‐derived HCC patient subgroups in PT and AT (david and MetaCore software). (A) david FA/GO heat map analysis (excluding 24 genes comprising the RGC) for the DEG subsets. Up‐regulated and down‐regulated DEGs in HR subgroups (HR_T_ and HR_A_) after RGC‐stratification in PT and AT and common for the Singapore and LCI cohorts. (B) Venn diagram and MetaCore cellular compartments heatmap analysis of tissue‐specific and common prognostic DEGs for PT and AT up‐regulated in HR_T_ and HR_A_ subgroups in the two studied cohorts. Gray cells: nonsignificant FA/GO terms. Color gradient: significant FA/GO terms [Benjamini corrected Fisher test –log_10_ *P*‐values (*P* < 0.05)].

The deregulated pathway associated with RGC‐defined HCC patient risk subgroups might be useful for designing new molecular targets and developing therapies for RGC‐stratified HCC patients. Using david software, the FA/GO enrichment analysis of the up‐regulated DEGs observed in the HR subgroups (HR_T_ and HR_A_) revealed FA/GO terms related to ribosomes, translation and the cell cycle in both the PT and AT, although the high enrichment of terms related to cytoplasmic vesicles was only observed in AT (Fig. 4A). The DEGs down‐regulated in the HR_T_ and HR_A_ subgroups displayed various common enriched terms, including deregulated liver metabolism [FA/GO terms ‘lipid metabolism,’ ‘carboxylic acid metabolic process’ (SP_PIR_KEYWORDS)] and mitochondrial dysfunction [e.g. FA/GO terms ‘GO:0005739~ mitochondrion’ and ‘oxidoreductase’ (SP_PIR_KEYWORDS)] (Fig. 4A).

Exclusively in PT (Fig. S7A), MetaCore Pathway Maps Analysis identified the deregulation of the genes involved in the WNT pathway (Fig. S7B). *DKK1*,* DVL3*, β‐catenin (*CTNNB1*), casein kinase II (*CSNK2A* and *CSNK2A2*) and *LEF1* were significantly up‐regulated, whereas GSK3‐β (*GSK3B*) was significantly down‐regulated (Fig. S8). These gene expression alterations are characteristic of *WNT* pathway deregulation (Tannock and Hill, 2013).

**Figure 5:**
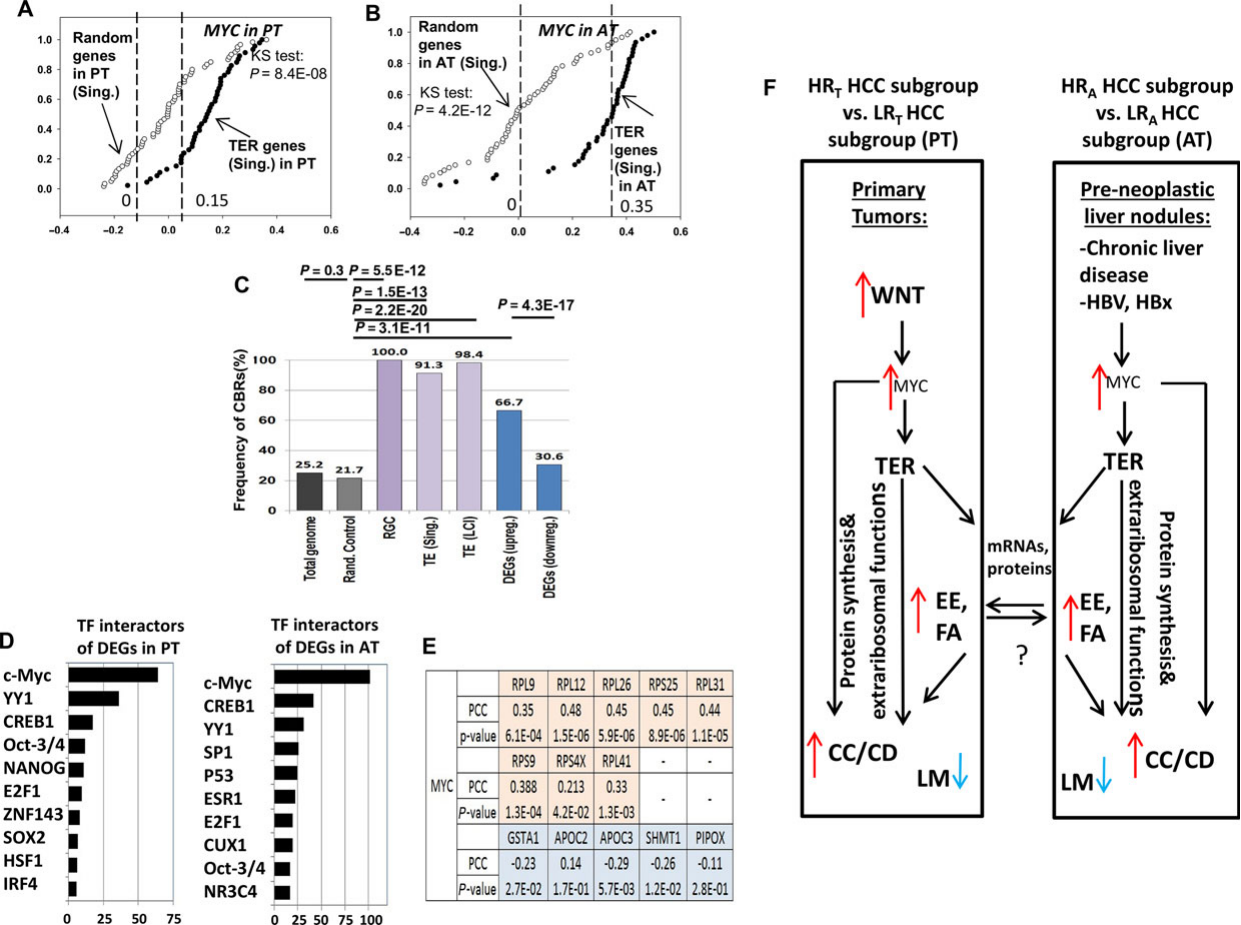
The computational analyses and hypothetical data‐driven model suggest the role of the TER pathway in PT and AT of HCC patients. (A, B) Correlation analyses of *MYC* in the Singapore and LCI cohorts in PT, AT. *x*‐axis: Kendall's Tau correlation coefficient; *y*‐axis: cumulative relative frequency. Black circles: correlation coefficients for TER gene sets (‘TER gene set (Singapore)’ and ‘TER gene set (LCI)’; white circles: correlation coefficients for random control gene set (see also the Supporting information: MYC as a key regulator of ribosomal pathway in HCC PT and AT). Dashed lines indicate medians for correlation coefficients distributions. (C) Frequencies of *MYC* ChIP‐seq binding regions in HepG2 cells in the vicinity of proximal promoters (+200/−500 bp) in TER gene sets and DEGs up‐regulated in HR_T_ subgroups. *x*‐axis: various gene sets; *y*‐axis: frequency of ChIP‐seq binding regions (%). Differences in the frequencies assessed using Fisher's exact test (see the Supporting information: MYC as a key regulator of ribosomal pathway in HCC PT and AT). (D) MetaCore transcription factors interactors significantly enriched for the DEGs after RGC stratification in PT and AT. *x*‐axis: −log_10_ transformation of FDR‐corrected *P*‐values (*P* < 0.05); *y*‐axis: MetaCore terms for transcription factor (TF) interactors. (E) Correlation analysis of MYC with eight representative TER genes and five genes involved in liver metabolism based on quantitative RT‐PCR data from 92 PT samples from the Singapore cohort. PCC, Pearson's correlation coefficient. (F) Hypothetical data‐driven model of the TER pathway in PT and AT of HCC patients**.** Red and blue arrows: genes up‐regulated and genes down‐regulated in HR HCC patient subgroups, respectively. TER, translation elongation/ribosomal genes; CC/CD, cell cycle/cell division genes; EE and FA, extracellular exosome and focal adhesion genes, respectively. LM, genes involved in liver metabolism.

*TGFBR2* and *MYC* were significantly overexpressed in the up‐regulated DEGs of both HR_T_ and HR_A_ patient subgroups (Tables S8 andS9). Our results, shown in Fig. 5 and Supporting information (section: MYC as a key regulator of ribosomal pathway in HCC PT and AT), provide the evidences that MYC is a key positive transcription regulator of the RGC and TER genes in PT and AT. MetaCore identified *MYC* as the most significant transcription factor interactor among the DEGs in the both PT and AT (Fig. 5D and Tables S8 and S9). Furthermore, combining gene expression correlation, FA/GO and ChIP‐seq analyses revealed that MYC might be the primary positive transcription regulator of the genes involved in the TER pathway (Fig. 5; Tables S3 and S10) in both PT and AT cells (Figs 5C and S9; see also the Supporting information: MYC as a key regulator of ribosomal pathway in HCC PT and AT).

MetaCore Pathway Map analysis suggested that DNA double‐strand break repair was characteristic of the PT but not AT (Fig. S7B). In both PT and AT, we identified the enrichment for cell cycle, cytoskeleton remodeling, and vascular endothelial growth factor and transforming growth factor‐β receptor signaling. The ‘GO localization’ category identified common GO terms for PT and AT for extracellular vesicles and exosomes, cell adherence junctions, focal adhesions and ribosomes (Fig. 4B), which suggested a deregulation of extracellular exosomal transport in the HR_T_ and HR_A_ HCC subgroups. For the PT‐specific gene subset, we found no association with extracellular vesicles. However, the AT‐specific subset was additionally associated with extracellular exosomes and endosomes. In the common PT and AT gene subset, 15 RGC genes were annotated under the MetaCore term ‘extracellular exosome’ (Fig. S7C). In the AT‐specific subset, at least eleven genes annotated under the term ‘endosome’ have previously been associated with endosome‐specific functions (Fig. S7C and Table S11). Within the up‐regulated AT‐specific DEGs subset, we identified multiple enriched Pathway Maps, indicating an activated immune response in the AT (Fig. S7B).

**Figure 6:**
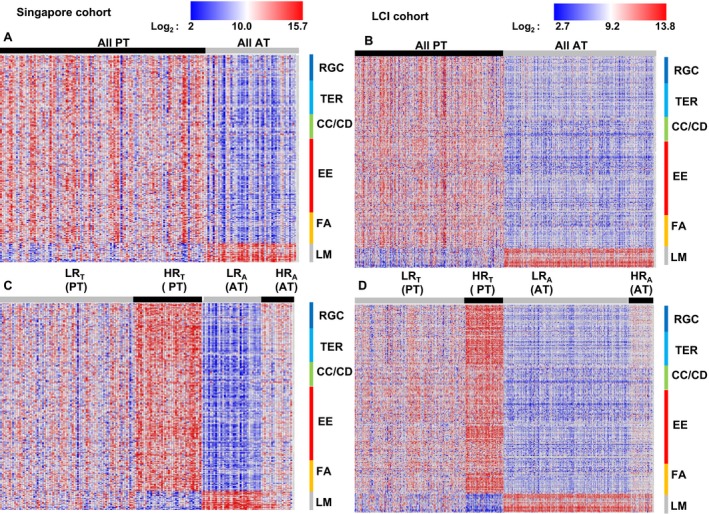
Gene expression heatmap for representative genes of common prognostic pathways in the nonstratified and RGC‐stratified PT and AT. The heatmap before (A, B) and after (C, D) stratification of HCC patients using the RGC. (A, C) Singapore cohort. (B, D) LCI HCC cohort. The pathways for the up‐regulated or down‐regulated DEGs in HR subgroups were selected as gene sets enriched under the specific FA/GO terms (Table S12). TER, genes enriched under the FA/GO term ‘GO:0006414~translational elongation’ in PT; CC/CD: cell cycle/cell division genes enriched under the FA/GO terms ‘GO:0007049~cell cycle’ and ‘cell division’ (SP_PIR_KEYWORDS); EE and FA, extracellular exosome and focal adhesion genes enriched under the ‘GO localization’ terms (MetaCore) ‘extracellular exosome’ and ‘focal adhesion’, respectively. LM, representative mitochondrial and oxidoreductase DEGs down‐regulated in HR subgroups in both PT and AT and enriched under the FA/GO terms ‘GO:0005739~mitochondrion’ and ‘oxidoreductase’. Mitochondria is known as an integrative energy hub of diverse liver metabolism pathways (Degli Esposti *et al*., 2012). Heat map spectrum displays log_2_ transformed gene expression values.

Importantly, the comparison between nonstratified PT and AT in the Singapore and LCI cohorts revealed global systematic differences in the gene expression profiles between these tissue types for common PT and AT prognostic pathways (Fig. 6A,B). However, after RGC‐based stratification, the representative common prognostic pathways (TER, cell cycle/cell division, extracellular exosome, focal adhesion and liver metabolism) (Table S12) displayed distinct characteristic gene expression patterns within the PT and AT (Fig. 6C,D).

### 3.7 Validation of the RGC and RGC‐associated genes using quantitative RT‐PCR in the Singapore cohort

We assessed the reproducibility of the gene expression measurements in the microarray platform with quantitative RT‐PCR experiments using RNA samples from the Singapore cohort. We selected eight representative ribosomal genes from the RGC, seven representative up‐regulated WNT pathway genes and five representative down‐regulated genes involved in liver metabolism in the HR_T_ subgroups in both cohorts. For the eight tested RGC genes (*RPL9, RPL12, RPL26, RPL31, RPL41, RPS9, RPS25* and *RPS4X*) and four tested up‐regulated WNT pathway genes (*MYC, ENC1, CTNNB1* and *LEF1*), we observed a strong and significant correlation with the microarray results (Fig. S10). We also tested the consistency in differential gene expression in these samples by comparing patients from the HR_T_ and LR_T_ subgroups originally classified using microarray gene expression data (Fig. S11). The quantitative RT‐PCR analysis indicated that the 15 up‐regulated genes from the HR_T_ subgroup (based on the microarray data, Fig. S11A,B) were up‐regulated in randomly selected patients from the HR_T_ subgroup compared to the LR_T_ subgroup (Fig. S11D); the five down‐regulated genes from the HR_T_ subgroup (based on the microarray data) (Fig. S11C) were down‐regulated in randomly selected patients from the HR_T_ subgroup compared to the LR_T_ subgroup (Fig. S11D).

Finally, the RGC stratification power using the quantitative RT‐PCR expression data was comparable to that of the microarray expression data (Fig. S11E versus Fig. 2C). In total, we analyzed PT from 92 HCC patients from Singapore cohort. The patients were selected at random. Patient's stratification was carried out according to the microarray identified RGC genes, detected in PT samples by the qRT‐PCR (Fig. 2C). The quantitative RT‐PCR based risk stratification analysis results (Fig. S11E) suggest the applicability of our prognostic model for quantitative stratification of the LR and HR HCC patients.

### 3.8 DKK1 is a potential prognostic factor and drug target

*DKK1* was found to be the most up‐regulated gene in the HR_T_ subgroups after whole transcriptome screening. We thus also tested whether *DKK1* gene expression was associated with the RGC‐based classification at the protein level in the Singapore HCC cohort. First, we selected six representative HCC patients from the Singapore cohort (three from HR_T_ and three from LR_T_ subgroups). Second, we analyzed liver tumor FFPE section images (Methods, Supporting information: Image based analysis of immunohistochemistry slides) using a training and cross‐validation protocol for the SVR system (Smola and Vapnik, 1997). We tested all possible pairs of the HR_T_ versus LR_T_ patients (nine pairs) by measuring the SVR scores of the corresponding tumor tissue images. As expected, the average predicted DKK1 SVR score of the LR_T_ patient images was significantly less than that of the HR_T_ patient images of the nine pairs (Fig. S12). We proposed DKK1 as a potential prognostic factor and drug target for a preclinical study of anti‐DKK1 therapy in HCC.

### 3.9 RGC‐based HCC stratification outperformed several known multigene HCC classifiers

The validation analyses of RGC in PT or AT HCC samples and univariate and multivariate analyses of the RGC suggest its robustness and reproducibility across HCC patient cohorts. We compared the prognostic power, robustness and reproducibility of the RGC with those of several published prognostic HCC multigene signatures. The comparison of the RGC with the 65‐gene risk signature (Kim *et al*., 2012), the 16‐gene G1–G6 signature (Boyault *et al*., 2007), the vascular invasion gene signature (Minguez *et al*., 2011) and the 186‐gene survival signature proposed for the analysis in AT (Hoshida *et al*., 2008) revealed the prognostic outperformance of the RGC in PT or AT in the cohorts under investigation (see Supporting information: RGC‐based HCC stratification performance). Briefly, in the combined overall HCC group, the first three gene signatures displayed nonsignificant prognostic power: 65‐gene risk signature, *P = *0.12, hazard ratio = 0.73 (95% CI = 0.50–1.09); the 16‐gene G1–G6 signature, *P = *0.3, hazard ratio = 1.24 (95% CI = 0.84–1.84); vascular invasion gene signature, *P = *0.3, hazard ratio = 1.27 (95% CI = 0.86–1.87) (not shown). The 5‐gene risk score signature proposed by Nault *et al*. (2013) was the only gene signature showing a significant prognostic value in PT, comparable with the RGC: 5‐gene risk score, *P = *3.1 × 10^−6^, hazard ratio = 2.57 (95% CI = 1.73–3.81) versus RGC, *P = *1.6 × 10^−6^, hazard ratio = 2.63 (95% CI = 1.77–3.92), respectively (Fig. S13A,B). Comparison of the patients stratified in the RGC‐derived HR_T_ subgroups with the HR patients, identified using the 5‐gene score signature in the two HCC groups, revealed substantial differences in their stratification: more than 50% of patients from HR_T_ subgroup were stratified using the 5‐gene score signature as a LR subgroup (Fig. S13C).

The RGC displayed stronger prognostic power compared to the 186‐gene survival signature in AT [RGC: *P = *4.7 × 10^−3^, hazard ratio = 2.01 (95% CI = 1.24–3.26); 186‐gene signature: *P = *0.01, hazard ratio = 1.77 (95% CI = 1.13–2.77)] (Fig. S13D,E). Interestingly, the combined use of the 186‐gene signature with the RGC substantially improved the prognostic power [Wald *P = *8.6 × 10^−4^, hazard ratio = 2.43 (95% CI = 1.42–3.87)] (Fig. S13F).

Finally, the RGC retained its prognostic power in multivariate analysis in PT compared to the 5‐gene risk score [RGC, *P = *3.2 × 10^−4^, hazard ratio = 2.15 (95% CI = 1.42–3.25] versus 5‐gene risk score signature [*P = *5.7 × 10^−4^, hazard ratio = 2.07 (95% CI = 1.37–3.14)] and in AT compared with the 186‐gene survival signature [RGC, *P = *0.02, hazard ratio = 1.80 (95% CI = 1.10–2.95)] versus 186‐gene survival signature [*P = *0.04, hazard ratio = 1.62 (95% CI = 1.02–2.56)] (Fig. S13G).

These results showed the superiority or independent prognostic power of the RGC compared to other proposed multigene prognostic signatures.

## 4 Discussion

Previous studies have identified the pathobiological pathways and clinically relevant HCC prognostic classifiers that utilized PT; PT with the AT serving as a negative control; or AT alone (Hoshida *et al*., 2008). However, increasing evidence indicates the presence of common co‐regulatory processes, signaling pathways and molecular mechanisms that drive PT and AT compartments (Polyak *et al*., 2009), resulting in their interconnection and complex regulatory patterns.

In the present study, we developed a prognostic stratification approach to identify common oncogenic pathways and survival significant prognostic variables in the PT and AT of HCC patients with resectable PT. This methodological approach revealed common co‐expression patterns for multiple PT‐ and AT‐associated TER pathway genes that are reproducibly associated with aggressive HCC. Further analysis suggested that this might reflect the pathophysiological mechanics of the TER pathway in HCC progression.

We identified 24‐ribosomal gene classifier (termed RGC) that displayed the significant PT‐associated prognostic power in the studied HCC patient cohorts. The RGC was clinically (Fig. 2C,D) and genetically (i.e. identical enriched biological gene sets) (Fig. 2E) reproducible in a large independent HCC patient dataset. The RGC retained its significant and independent prognostic power in a multivariate analysis compared to the traditional clinicopathological parameters of HCC (Table 3). A comparison of RGC with the 65‐gene risk signature (Kim *et al*., 2012), the 16‐gene G1–G6 signature (Boyault *et al*., 2007) and the vascular invasion gene signature (Minguez *et al*., 2011), previously proposed for prognosis in PT, demonstrated the outperforming prognostic power of the RGC. The RGC displayed comparable and independent prognostic potential (i.e. clinical reproducibility) compared to the 5‐gene score signature of Nault *et al*. (2013) (Fig. S13A–C), which was also developed for HCC patients with curative resection. However, in contrast to the RGC, the genetic reproducibility of the 5‐gene score signature remains unclear because it has not been tested (Nault *et al*., 2013). Notably, the alternative proliferative 41‐gene cell cycle gene signature generated from DEGs between PT and AT failed in cross‐cohort validation (Fig. S6), indicating the limitations of cell cycle‐based DEG approach(s) for generating a reproducible HCC prognostic classifier.

RGC could be considered for prognosis using gene expression information not only from PT, but also from AT in the same HCC patients (Fig. 4A–B). Comparison of the RGC with the 186‐gene survival signature, previously developed for prognosis in AT (Hoshida *et al*., 2008), revealed a stronger and independent prognostic value for the RGC in the studied cohorts (see 2; Fig. S13; see also the Supporting information: RGC‐based HCC stratification performance). Thus, the RGC could potentially be used for pre‐surgery prognostic patient stratification using AT biopsy tissue samples. The AT‐based RGC stratification could minimize the risks of post‐biopsy tumor dissemination and intrahepatic metastasis. Interestingly, the combined use of the RGC with the 186‐gene survival signature (Fig. S13F) or the RGC alone in both PT and AT (Fig. 3D) can significantly improve the post‐surgical prognostic power of the RGC.

The tumor suppressor genes *FBP1* and *SPOP* and their products acting as CPGs in HCC livers may also be important diagnostic and prognostic biomarkers and therapeutic targets.

The data analysis of DEGs in the RGC‐derived HR patient subgroups in the PT revealed the deregulation of key *WNT* pathway‐associated genes, including *DKK1*,* MYC, CTNNB1* and *LEF1*. Therefore, the RGC prognostic stratification in the PT reflects the deregulation of the upstream regulatory molecular machinery towards the *WNT*‐β‐catenin‐*MYC* axis.

*DKK1* has previously been demonstrated as a promising diagnostic and prognostic factor of HCC (Shen *et al*., 2012; Yu *et al*., 2009). After RGC‐based stratification, *DKK1* was the most up‐regulated DEG between the HR_T_ and LR_T_ subgroups (PT) in both LCI and Singapore cohorts (Table S8) and was found to be a pro‐oncogenic prognostic factor (Fig. S12A,B). This finding was validated using quantitative RT‐PCR (Fig. S11), strongly confirming the results of previous reports. Additionally, quantitative *DKK1* immunostaining demonstrated a significant association between *DKK1* overexpression and the RGC classification (Fig. S12; see the Supporting information: Image based analysis of immunochemistry slides). Because DKK1 has been demonstrated to be a pathologically essential and strong prognostic factor in several studies, including ours, *DKK1* could be a potential candidate for a future preclinical study of anti‐*DKK1* therapy in HCC, as described for myeloma (Fulciniti *et al*., 2009) and osteosarcoma (Goldstein *et al*., 2016).

The results of the present study suggest that the subset of TER pathway genes (including the RGC genes) and multiple DEGs associated with alterations of this pathway might simultaneously be controlled by *MYC* in the PT and AT cells of HCC patients (Fig. 5A–E). By contrast to PT, we did not observe a significant enrichment of deregulated WNT pathway genes in AT (Fig. S7B), indicating that other factors, such as progression of viral HBV and/or chronic liver disease (Chan *et al*., 2004), may deregulate AT‐associated MYC expression and TER pathway genes during HCC progression. Interestingly, the HBx HBV viral protein can cooperate with *MYC* to support ribosome biogenesis in HCC cell lines (Shukla and Kumar, 2012).

CPG‐based prognostic biomarker preselection resulted in the detection of enriched FA/GO terms, where the enrichment for TER genes substantially predominated over the enrichment for cell cycle/cell division genes (Figs 1F and S4; see also the Supporting information: *MYC* as a key regulator of ribosomal pathway in HCC PT and AT).

Furthermore, we found that HR patient HCC subgroups after RGC stratification were characterized by a predominance of up‐regulated TER genes and a subset of down‐regulated liver metabolism genes (Fig. 4). In this respect, an interesting study (Coulouarn *et al*., 2006) described a comparison of the global gene expression profiles of liver pre‐neoplastic nodules in three transgenic mouse models with the over‐expression of *c‐Myc* alone, *E2f1* alone, and both *E2f1/c‐Myc*, respectively. Interestingly, the gene expression profiles for the liver pre‐neoplastic nodules detected in the *c‐Myc* over‐expression mouse model were specifically characterized by induction of the genes involved in protein synthesis and the repression of the genes regulating liver metabolism. Noteworthy, the gene expression patterns observed in HR_T_ and HR_A_ subgroups (Fig. 4) displayed a remarkable similarity with that of the transgenic Myc mouse model of pre‐neoplastic liver nodules. We suggest that the results and RGC‐based prognostic model developed in the present study probably reflect the essential pathobiological processes in early‐stage MYC‐driven malignization in AT liver cells, which consequently affect tumor progression and poor OS of HCC patients.

Recent studies support this notion, highlighting many examples of the tumorigenic extra‐ribosomal functions of the genes involved in translation (Kim *et al*., 2004; Wang *et al*., 2015b) and their active and important roles in cell cycle regulation and apoptosis (Stumpf *et al*., 2013). At certain stages of tumorigenesis, TER pathway genes could play a role in pro‐oncogenic dysregulation, malignization and cancer progression in liver tissue. In this context, uncontrolled cell cycle and cell division, as major features of malignancy and progression drivers distinguishing PT from AT (Fig. 1F), may be considered just as another component of the entire pathobiology process.

Details of the molecular mechanisms underlying the co‐expression of pro‐oncogenic TER genes in PT and AT are not understood; this could be the subject of future studies. However, this does not exclude the possibility that the TER (and many other RGC‐associated genes, some of which were described and considered in the present study) is responsible for the cross‐tissue interactions between PT and AT during HCC progression. For example, *TGFBR2*, a key extracellular signaling regulator in multiple cancers (Morris *et al*., 2012), was significantly overexpressed in the HR RGC subgroups in both the PT and AT. The TER pathway genes were significantly associated with genes encoding extracellular vesicular and exosomal components, focal adhesions and adherence junctions (Fig. 4), assuming the hypothetical transfer of ribosomal mRNAs and proteins between the PT and AT as the potential cellular reprogramming/malignization factors. Notably, the mRNAs of multiple TER pathway‐associated genes and rRNAs are often abundant in microvesicles/exosomes secreted by cancer cells (Jenjaroenpun *et al*., 2013; Van Deun *et al*., 2014).

Thus, we propose the HCC‐liver tissue interaction model (Fig. 5F) in which the up‐regulation of ribosomal CPGs expression could support tumor cell growth and proliferation in PT by enhancing protein synthesis and additionally inducing similar ribosome biogenesis pathways in AT via the extra‐ribosomal functions. In addition, CPG products could be involved in tumorigenic signaling pathways through activated extracellular exosomal trafficking (Fig. 5F).

The HR patient subgroups identified using the RGC in PT and AT (Figs 2 and 3) would have wide‐ranging targeted treatment options throughout a single molecular cascade (Figs 4 and S8), thus enabling the further identification of novel and efficient drugs for patient subgroups with poor clinical outcomes (see also the Supporting information: Potential therapeutic intervention strategies after RGC‐based HCC risk stratification). Finally, the simultaneous targeting of common prognostic genes and pathways in PT and AT may be considered as an alternative approach for potentially reducing post‐surgery disease relapse.

The RGC stratification is comparatively simple (24 prognostic genes) and robust, yielding biologically consistent and reproducible patient stratification (Fig. 2) with a thoroughly characterized pathway deregulation (Figs 4 and S8) for use as a practical HCC prognostic assay.

There are limitations associated with the present study. First, the tissues and clinical data were collected retrospectively. Thus, this prognostic model needs further validation in a prospective study. Second, the present study only included HCC patients who underwent surgical resection; they are a selected group and some of our results may be not automatically extrapolated to other clinical HCC groups. For example, the RGC was cross‐cohort validated in the LCI cohort, which predominantly includes HBV‐related HCC patients (91%) with liver cirrhosis (92%); the RGC prognostic utility for non‐HBV‐related HCC patients and/or those without liver cirrhosis needs to be confirmed in another study.

In summary, our predictor selection and survival prediction analysis identified the ribosome biogenesis genes co‐expressed in PT and AT from 321 HCC patients. The 44 TER CPGs could be considered as perspective HCC malignancy and prognostic biomarkers and targets for therapeutic implementations. We proposed that the PT‐inducted ribosomal biogenesis associated with the activation of TER pathways leads to extracellular signaling and ‘assimilation’ of a pro‐tumorigenic state in AT cells. In PT and AT, we introduced the 24‐ribosome gene‐based prognostic classifier suggesting a pathophysiological role of the ribosome biogenesis in both tissues.

## Author contributions

VAK, PC, OG and SPY were responsible for the study concept and design. PC and KL were responsible for acquisition of clinical and immunohistochemistry data. PC was responsible for liver tissue samples. KHL was responsible for quality control and verification of histological data. MS, HKL, SPY, OG and VAK were responsible for immunohistochemistry quantitative image analysis and its interpretation. OG and VAK were responsible for statistical and bioinformatics analyses. VAK, OG, SPY and PC were responsible for experimental design. SPY and RI were responsible for RNA sample collection, microarrays production and the wet‐lab study. OG, SPY, HKL, PC and VAK were responsible for the discussion of the experimental results and their interpretation. OG, VAK, SPY and MS were responsible for the drafting of the article. OG and VAK were responsible for the final version of the manuscript submitted for publication.

## Ethics statement

SingHealth has approved this study centralized Institutional Review Board. Protocol title: An integrative genomic approach for molecular classification of HCC and correlations with the clinical outcomes. Institutional Review Board Ref: 2013/788/B.

## Data accessibility

Research data pertaining to this article have been deposited at figshare.com: https://dx.doi.org/10.6084/m9.figshare.5616250.

## Supporting information


**Fig. S1.** 1D‐DDg method stratifies patients onto LR and HR subgroups based on ‘tumor suppressor‐like’ and ‘pro‐oncogenic’ prognostic gene expression patterns.
**Fig. S2.** Workflow of data analysis and validation.
**Fig. S3.** Survival analysis for RPL3 (ribosomal protein L3) and SPOP (speckle‐type POZ protein) CPGs.
**Fig. S4.** FA/GO analysis of TER genes using panther bioinformatics software.
**Fig. S5.** Identification of TER gene set in the LCI cohort and comparison with the TER gene set from the Singapore cohort.
**Fig. S6.** Training and cross‐cohort validation of the 41‐gene cell cycle gene signature.
**Fig. S7.** Up‐regulated DEGs after RGC‐stratification: MetaCore Pathway Maps and GO localization analyses.
**Fig. S8.** Deregulated WNT pathway in HR_T_ subgroups revealed after the RGC stratification of HCC patients in Singapore and LCI cohorts.
**Fig. S9.** *MYC* as a key regulator of genes involved in TER pathway in HCC PT and AT.
**Fig. S10.** Concordance analysis between expression data from microarray and quantitative RT‐PCR experiments in 12 representative genes.
**Fig. S11.** Quantitative RT‐PCR validation of 20 representative genes either involved in the RGC or genes differentially expressed in HR_T_ and LR_T_ subgroups.
**Fig. S12.** Results of the testing of *DKK1* relative expression in IHC liver tumor tissue images (PT) in six representative HCC patients using the SVR approach.
**Fig. S13.** Comparison of RGC prognostic power with other prognostic gene signatures.

**Table S1.** The flow of HCC patients and variables used in the present study.
**Table S2.** Checklist of REMARK guidelines in the present study.
**Table S3.** GO term ‘Translational elongation’ genes and their prognostic characteristics in Singapore dataset.
**Table S4.** GO term ‘Translational elongation’ genes and their prognostic characteristics in LCI dataset.
**Table S5.** The 24‐ribosome gene prognostic classifier and the 1‐D DDg‐derived characteristics used in validation analysis.
**Table S6.** 41‐gene prognostic signature defined by the cell cycle genes differentiating PT and AT samples. Data include Illumina and Affymetrix microarray probes annotation support.
**Table S7.** Clinical phenotypes associated with the RGC clinical subgroups in PT.
**Table S8.** The common differentially expressed genes between HR_T_ and LR_T_ prognostic subgroups derived by the RGC in PT.
**Table S9.** Common differentially expressed genes between HR_A_ and LR_A_ prognostic subgroups derived by the RGC in AT.
**Table S10.** Unique overlaps of ChIP‐seq binding regions with gene proximal promoters in various gene sets.
**Table S11.** Literature support of the selected genes involved in endosome functions.
**Table S12.** Common differentially expressed genes in HR RGC prognostic subgroups in both PT and AT.
**Table S13.** Oligoprimers used for quantitative RT‐PCR validation of the selected genes.
