# Supplementary material for: Tumor‐adjacent tissue co‐expression profile analysis reveals pro‐oncogenic ribosomal gene signature for prognosis of resectable hepatocellular carcinoma
Source: Mol Oncol. 2017 Dec 12;12(1):89–113. doi: 10.1002/1878-0261.12153 (PMC5748488; doi:10.1002/1878-0261.12153)
Supplement: Supplementary file 1 — Fig. S1. 1D‐DDg method stratifies patients onto LR and HR subgroups based on ‘tumor suppressor‐like’ and ‘pro‐oncogenic’ prognostic gene expression patterns. Fig. S2. Workflow of data analysis and validation. Fig. S3. Survival analysis for RPL3 (ribosomal protein L3) and SPOP (speckle‐type POZ protein) CPGs. Fig. S4. FA/GO analysis of TER genes using panther bioinformatics software. Fig. S5. Identification of TER gene set in the LCI cohort and comparison with the TER gene set from the Singapore cohort. Fig. S6. Training and cross‐cohort validation of the 41‐gene cell cycle gene signature. Fig. S7. Up‐regulated DEGs after RGC‐stratification: MetaCore Pathway Maps and GO localization analyses. Fig. S8. Deregulated WNT pathway in HRT subgroups revealed after the RGC stratification of HCC patients in Singapore and LCI cohorts. Fig. S9. MYC as a key regulator of genes involved in TER pathway in HCC PT and AT. Fig. S10. Concordance analysis between expression data from microarray and quantitative RT‐PCR experiments in 12 representative genes. Fig. S11. Quantitative RT‐PCR validation of 20 representative genes either involved in the RGC or genes differentially expressed in HRT and LRT subgroups. Fig. S12. Results of the testing of DKK1 relative expression in IHC liver tumor tissue images (PT) in six representative HCC patients using the SVR approach. Fig. S13. Comparison of RGC prognostic power with other prognostic gene signatures. [file MOL2-12-89-s001.pdf]

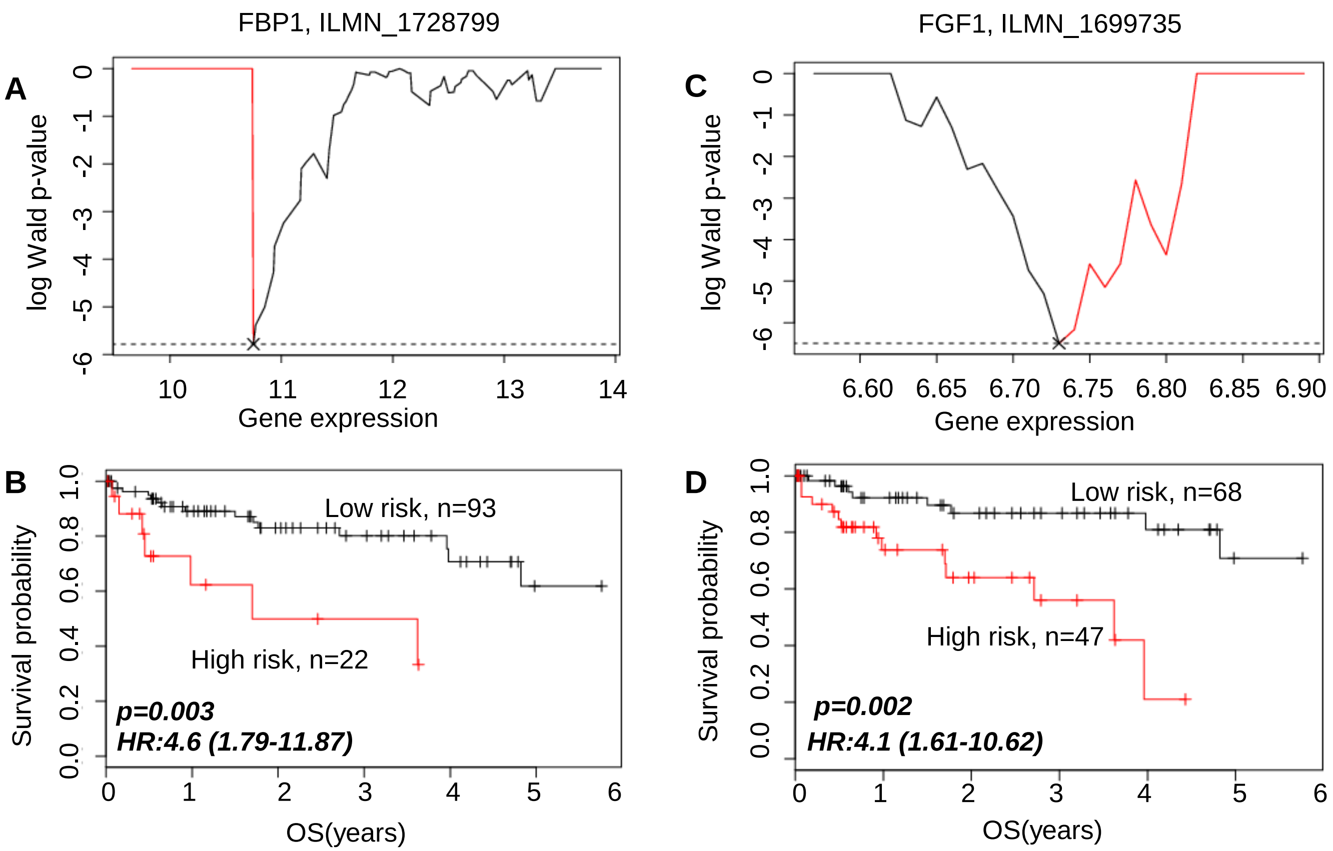

Figure S1. 1D-DDg method stratifies patients onto low-risk (LR) and high risk (HR) subgroups based on "tumor suppressor-like" and "pro-oncogenic" prognostic gene expression patterns. (A-B) Fructose-1,6-bisphosphatase 1, FBP1, a gluconeogenesis regulatory enzyme, acting as a rate-limiting enzyme in gluconeogenesis and known as a tumor suppressor and a key regulator of glycolysis. (C-D): Fibroblast growth factor 1, FGF1. (A) and (C): The p-value potential function of the goodness-off split 1D DDg analysis plot [25]. X-axis: gene expression level of the samples used for the identification of the group splitting cut-off value. Y-axis: natural log (*p*-values). The p-value of the Wald statistics test for the Kaplan-Meier survival curves was calculated. Black cross indicates the minimal Wald p-value (dotted line) corresponding to the 1D-DDg optimal gene expression cut-off value. (B) and (D): Kaplan-Meier survival plots defined by the 1-D DDg selected cut-off values. (B) and (D): X-axis: survival probability; Y-axis: Overall survival time (years). Red and black the survival curves denote HCC patient subgroups with relatively high- and low- risks of the disease outcomes, correspondingly. Singapore cohort's HCC samples were analyzed.

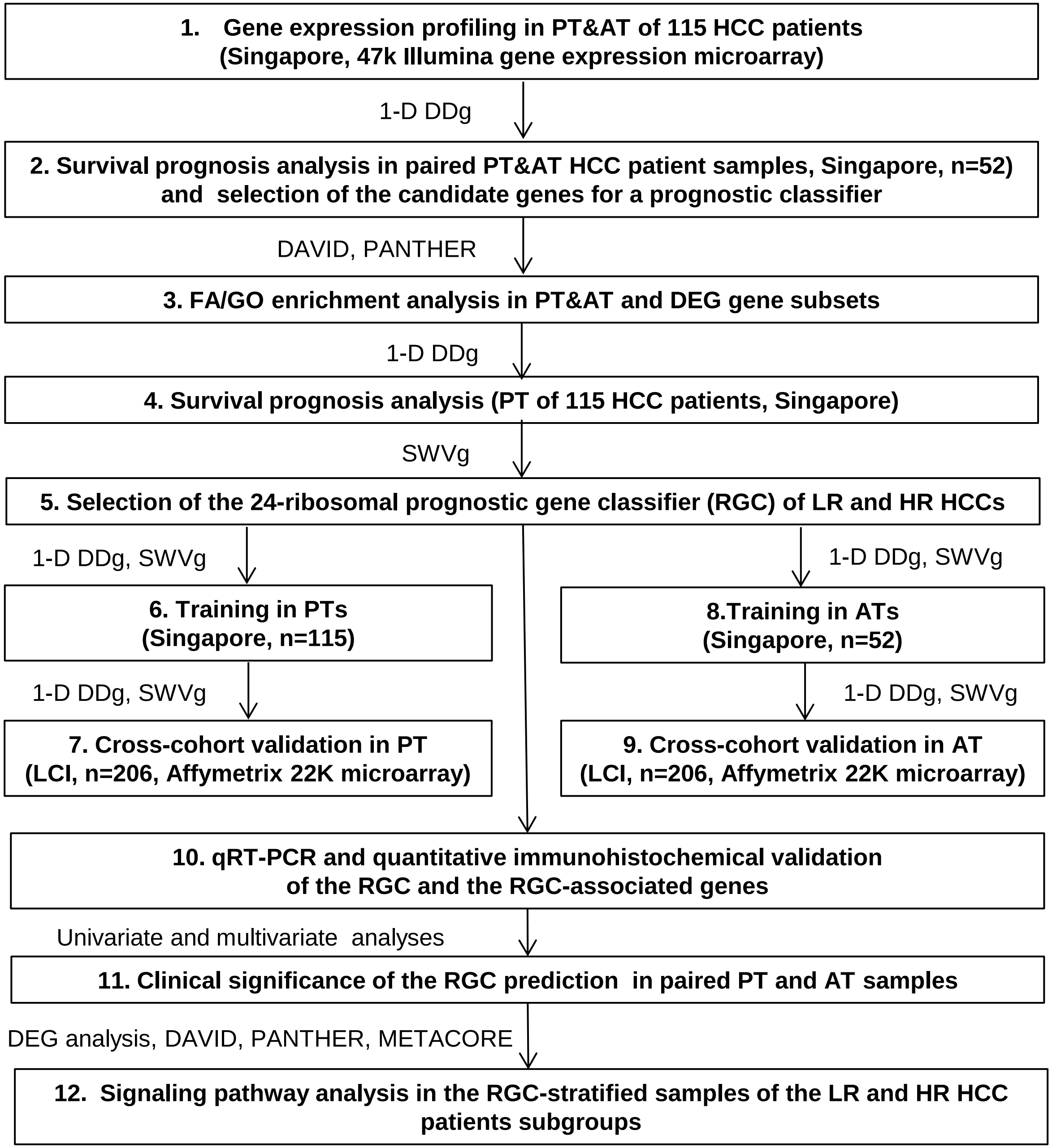

Figure S2. Workflow of data analysis and validation.

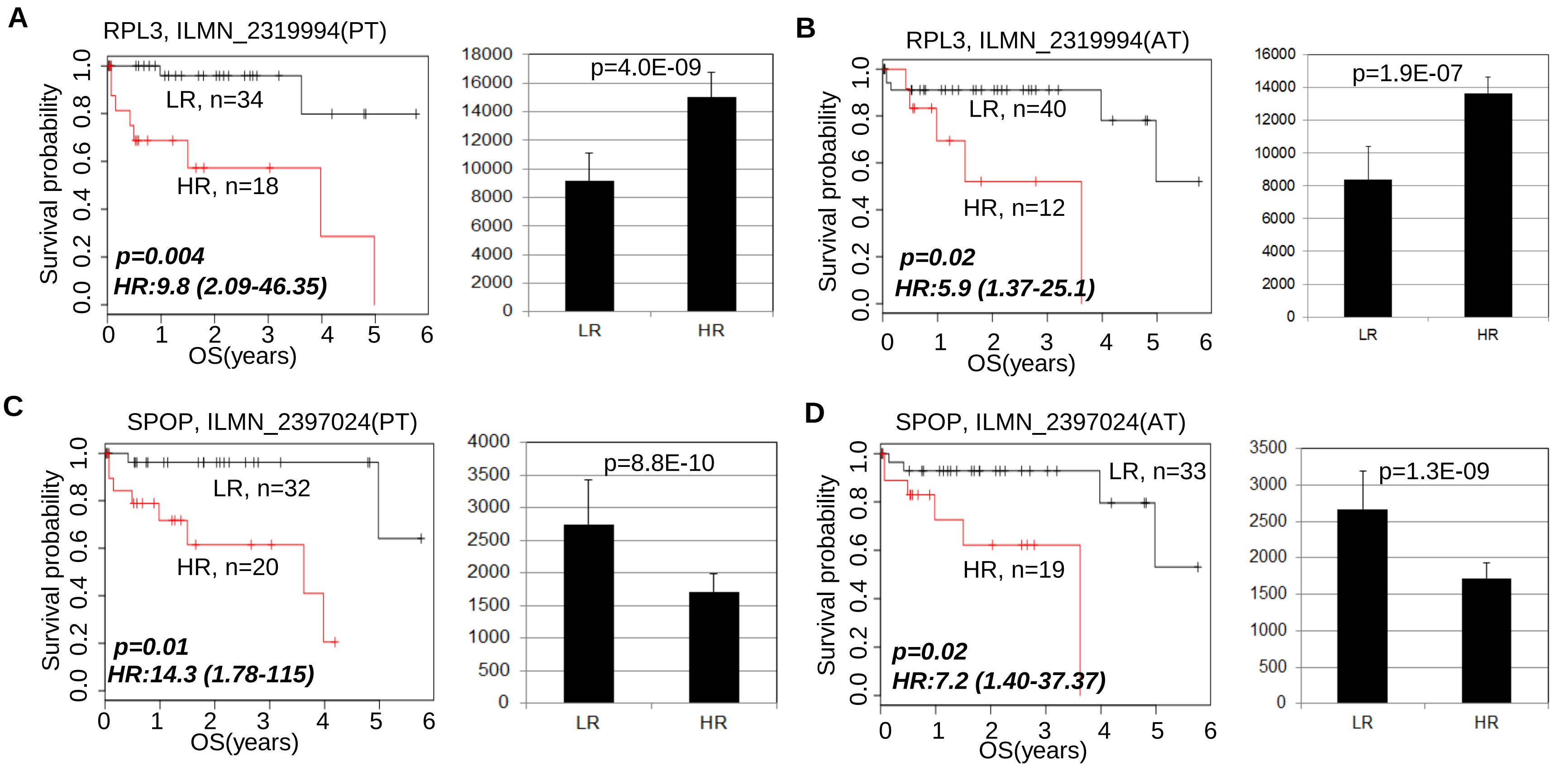

Figure S3. Survival analysis for RPL3(ribosomal protein L3) and SPOP (speckle-type POZ protein) CPGs.
(A), (B), (C) and (D) Kaplan-Meier curves obtained by 1-D DDg using the fitting of expression values to survival data. Analysis was performed independently in PT and AT for each gene in the Singapore (n=52) HCC cohort. Vertical bars and p-value show the significant difference in the level of gene expression between the low- and high-risk patient subgroups (Mann-Whitney test).

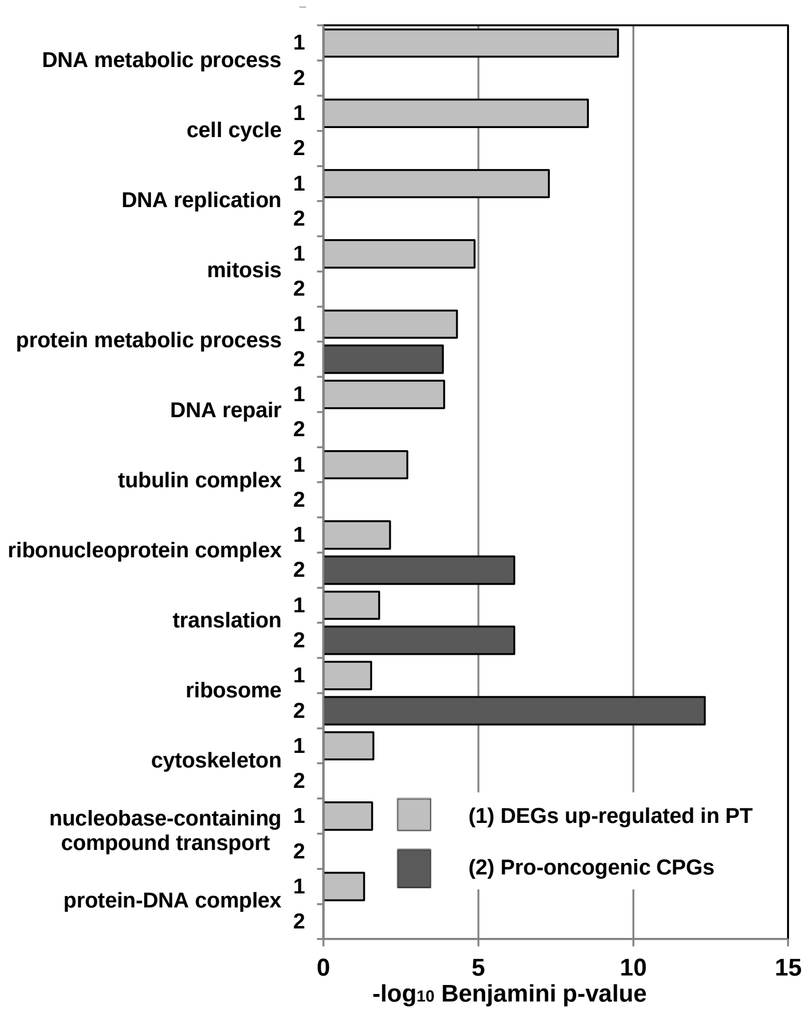

Figure S4. FA/GO analysis of TER genes using PANTHER bioinformatics software. FA/GO enrichment analysis for **(1)** the subset of top 1000 DEGs significantly up-regulated in PT compared to AT, and **(2)** the top 1000 pro-oncogenic CPG subset. Only highly significant representative FA/GO terms are shown. Fisher test *p*-values ($p<0.05$) at Benjamini correction in the $-\log_{10}$-transformed scale. Singapore cohort TER genes were studied.

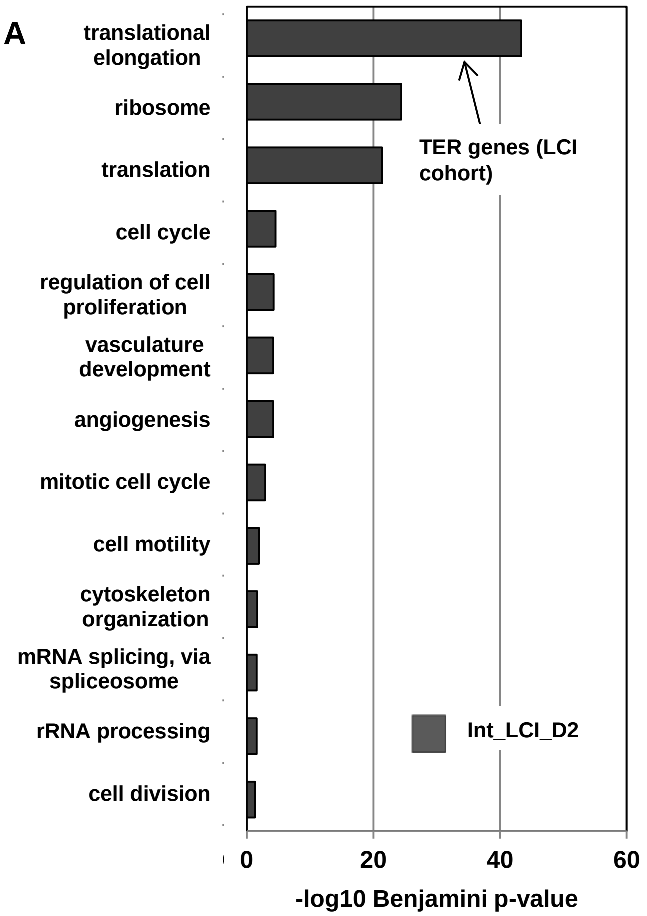

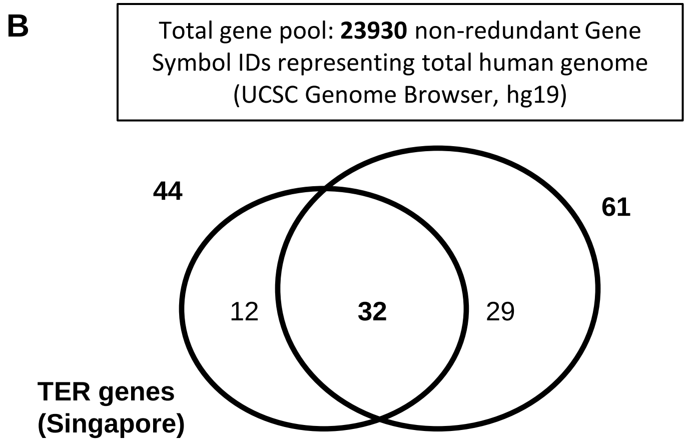

Hypergeometric test:	
	Cumulative probability:
TER genes(Singapore)/ TER genes (LCI)	**P(x≥32)=1.5E-80**

Figure S5. Identification of TER gene set in the LCI cohort and comparison with the TER gene set from the Singapore cohort. Identification of pro-oncogenic CPGs in the LCI HCC cohort using the 1-D DDg method for survival prognostic analysis was done exactly as it was done in the Singapore cohort (see Results). In the output of the workflow, only the GPG subsets having the identical 1-D DDg design 2 (pro-oncogenic CPGs, see Methods) in both PT and AT have been selected. (A) FA/GO enrichment analysis for the "pro-oncogenic" CPGs subset (1-D DDg design 2, "Int_LCI_D2”). "Int_LCI_D2": “pro-oncogenic” GPG subset with the identical 1-D DDg design 2 in both PT and AT. Only the highly significant representative FA/GO terms are shown (Fisher test Benjamini corrected p-values, p<0.05). (B): Enrichment analysis between TER gene sets (Singapore vs. LCI HCC cohorts, hypergeometric test; see also Tables S3 and S5).

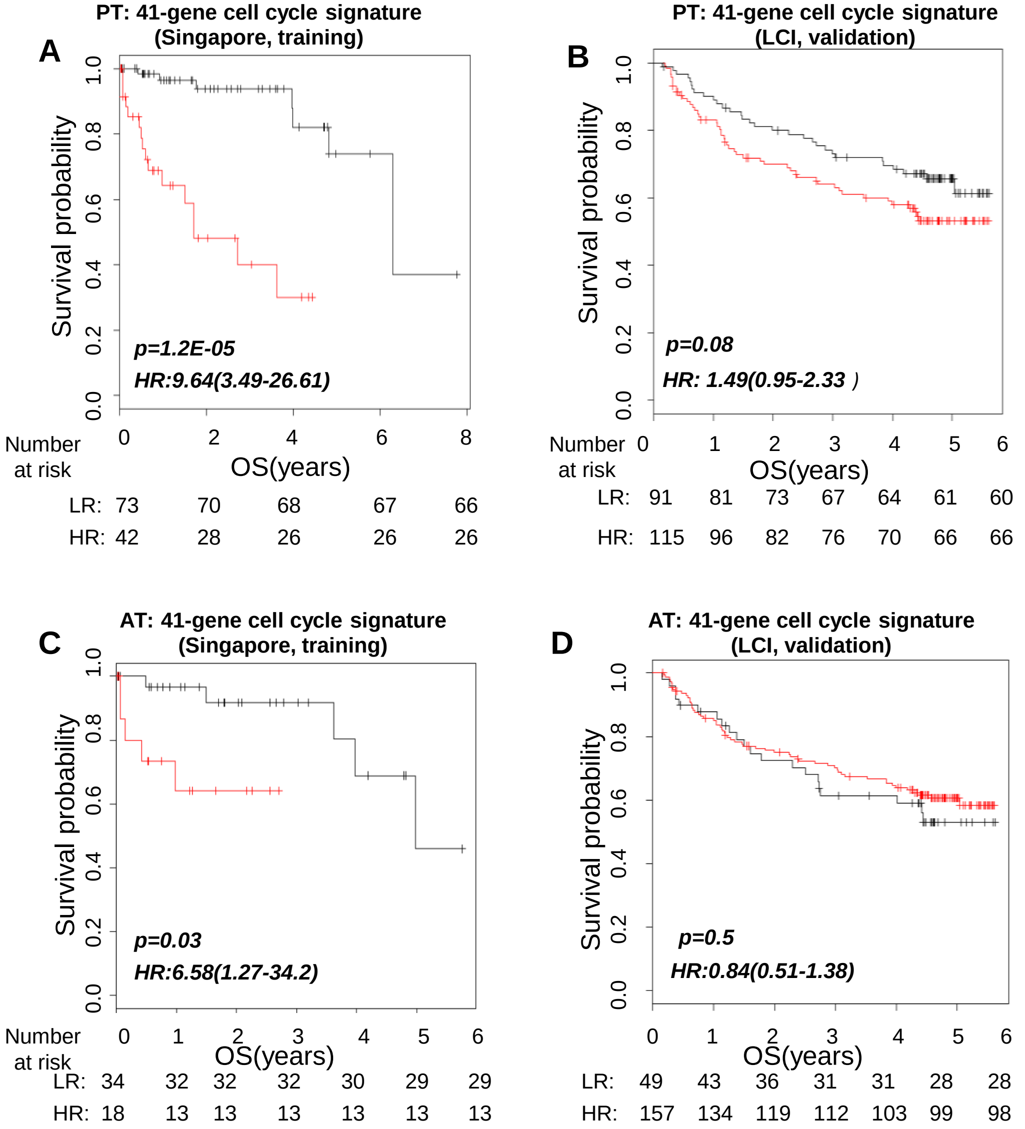

Figure S6. Training and cross-cohort validation of the 41-gene cell cycle gene signature. Kaplan-Meier survival curves for the LR and HR subgroups in the Singapore and the LCI cohorts. X-axis: survival probability; Y-axis: Overall survival (years). LR: low risk, HR: high risk. (A) and (B): training and cross-cohort validation of the cell cycle gene signature in PT samples. (C) and (D) training and cross-cohort validation of the cell cycle gene signature in AT samples.

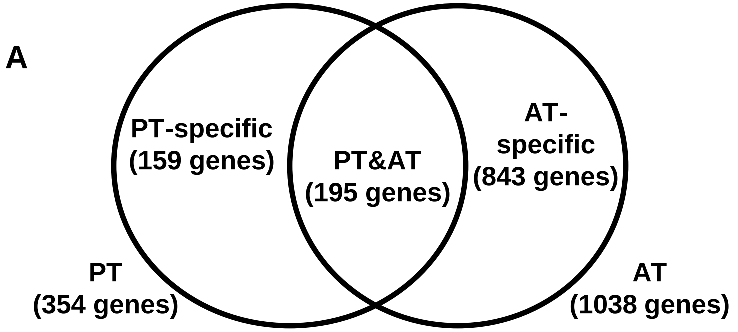

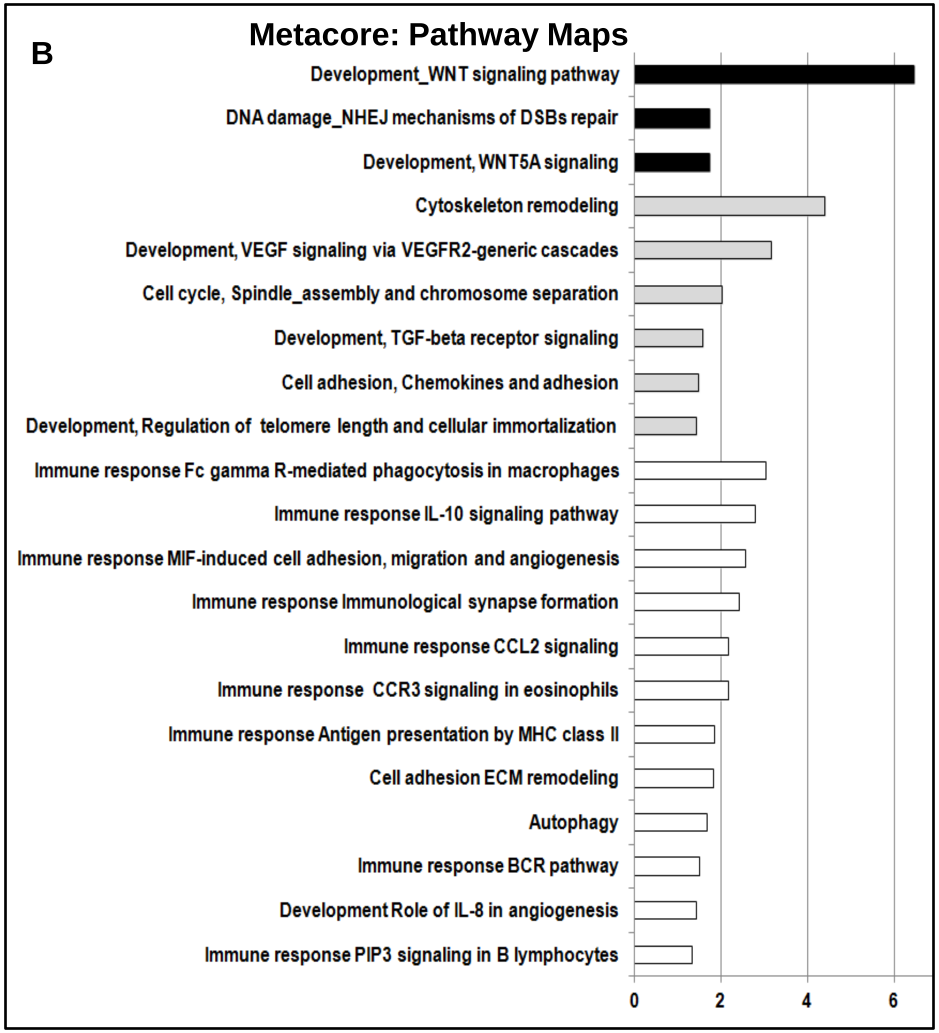

C

"extracellular exosome" (PT&AT intersec. gene set, see highlight in Fig. 4B)

ACTG1	CS	IARS	RNF103	RPL35A	RPS29	TNPO1
ADAM10	CTNNB1	ITGB1BP1	RPL10A	RPL37A	RPS3	TRMT112
ADSS	CTSC	MAPK1	RPL12	RPL7	RPS3A	TUBB6
ARF4	DNAJC5	MAPK3	RPL15	RPL7A	RPS4X	UBC
ARPC3	DYNLL1	MARCKS	RPL22	RPS11	RPS5	UFC1
ATP1B3	EEF1A1	MOB1A	RPL23	RPLP0	RPS7	VPS26A
CACYBP	EEF1G	NACA	RPL23A	RPS13	RPS8	WDR1
CCT2	EIF3H	NCKAP1	RPL24	RPS14	RPS9	YWHAZ
CCT6A	FABP5	NCL	RPL26	RPS15A	RPSA	ZNHIT6
CCT7	FKBP1A	PDCD2	RPL27	RPS17	SEC11A	
CD2AP	GLRX3	PPA1	RPL3	RPS18	SLK	
CHMP3	GYG1	PTTG1IP	RPL30	RPS2	STMN1	
CLIC1	HNRNPA1	RAN	RPL31	RPS25	TCP1	
CLN5	HSPA8	RBMX	RPL34	RPS27A	TMEM109	

"endosome"(AT specific gene set, see highlight in Fig. 4B)

APP	DARC	LGMN	RHOA	UBE2D3
ATP1A1	APPL2	MMD	S1PR1	UVRAG
ANXA1	DERL1	NIPA2	SCAMP1	ATP6V0E1
ANXA2	VOPP1	F2R	SNX3	VAMP3
CD74	FCGR2A	PLEKHB2	SNX7	VAMP5
ACAP2	ARL8B	PLEKHF2	STAM	VPS28
CHMP1B	HBXIP	PTP4A3	SHC1	VPS35
CHMP2B	HLA-DQA1	PFN1	STX6	WASF2
CHMP5	HLA-DRA	RAB32	STX8	
CALCRL	HLA-DPA1	RAB11FIP1	TMEM165	
CXCR4	IRAK1	RAB20	TPT1	
CTSL1	ITGB1	RAC1	CAP1	
CAV1	KIAA0196	RRAGA	TSG101	
GJA1	LAMP2	RRAGD	TUBA1A	

Figure S7. Up-regulated DEGs after RGC-stratification: MetaCore Pathway Maps and GO Localization analyses. (A) Venn diagram (top) and (B) Pathway Maps analysis (bottom) of tissue-specific (black: PT-specific; white: AT-specific) and common (grey) prognostic DEGs for PT and AT up-regulated in HR_T and HR_A subgroups in two studied cohorts. Only significant FA/GO terms (Benjamini corrected Fisher test -log10 p-values<0.05) are presented. (C) Gene symbol lists annotated under the "GO Localization" terms "extracellular exosome" and "endosome" (Fig.4B). Grey highlight: gene symbols of the RGC, blue highlight: gene symbols of representative genes involved in endosome-specific functions.

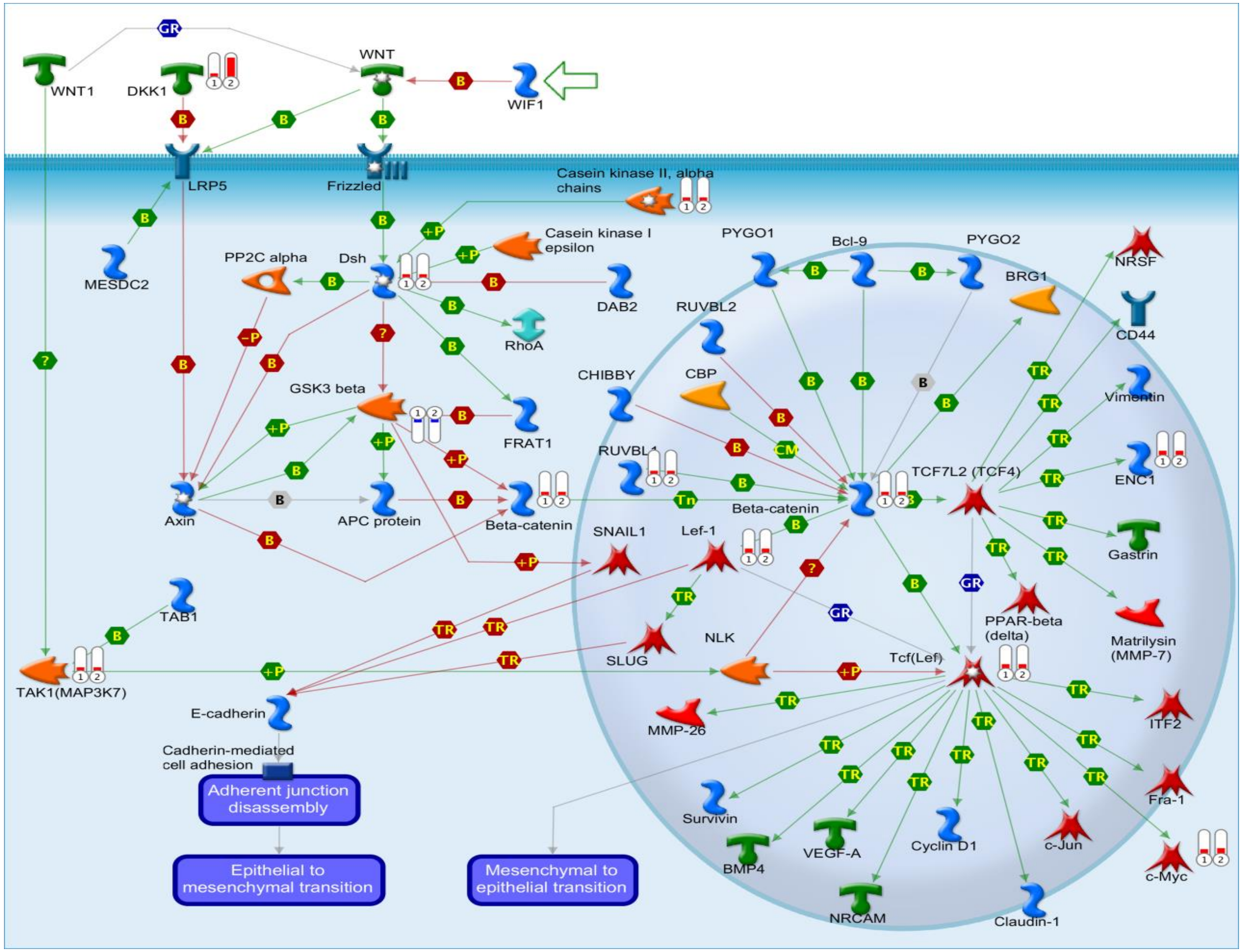

Figure S8. Deregulated WNT pathway in HR_T subgroups revealed after the RGC stratification of HCC patients in Singapore and LCI cohorts

(A) DEGs deregulated in HR subgroups and involved in WNT signaling pathway in the Singapore (bar 1) and the LCI (bar 2) cohorts (321 HCC patients analyzed). Numbers in the bottom table indicate the gene expression fold change in HR_T compared to LR_T subgroups in both cohorts (red: up-regulated in HR_T, blue: down-regulated in HR_T). Gene expression differences were significant for all genes in both cohorts (t-test q-value<0.05).

Fold change of gene expression for deregulated genes of WNT pathway in HR_T vs. LR_T RGC subgroups

	DKK1	DVL3	GSK3B	CTNNB1	CSNK2A1
**Singapore (1)**	*1.4*	*1.2*	*-1.2*	*1.3*	*1.2*
**LCI (2)**	*7.9*	*1.1*	*-1.3*	*1.2*	*1.3*
	**LEF1**	**MYC**	**ENC1**	**RUVBL1**	
**Singapore (1)**	*1.4*	*1.7*	*1.4*	*1.2*	
**LCI (2)**	*1.5*	*2.0*	*1.6*	*1.3*	

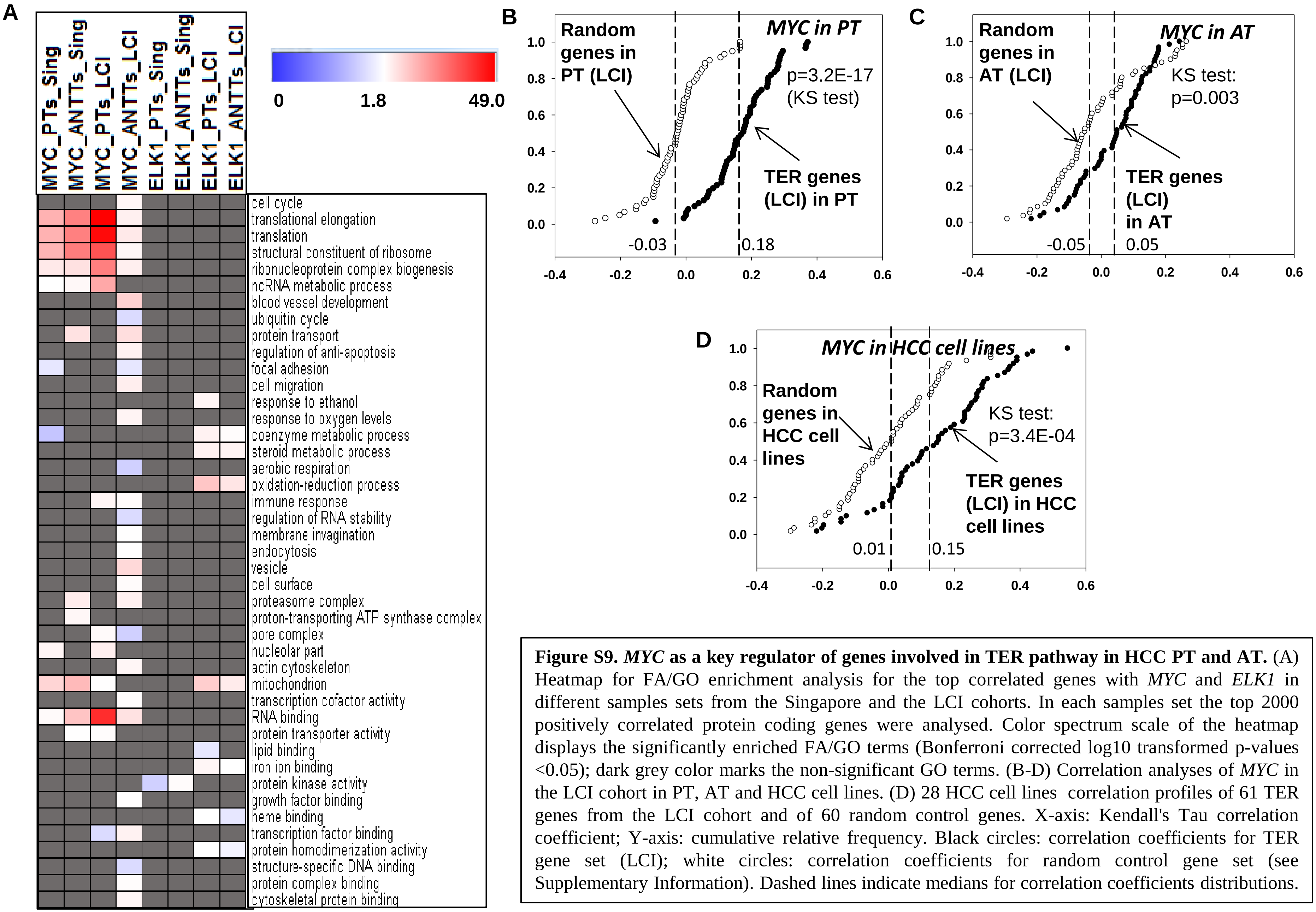

Figure S9. ***MYC*** **as a key regulator of genes involved in TER pathway in HCC PT and AT.** (A) Heatmap for FA/GO enrichment analysis for the top correlated genes with *MYC* and *ELK1* in different samples sets from the Singapore and the LCI cohorts. In each samples set the top 2000 positively correlated protein coding genes were analysed. Color spectrum scale of the heatmap displays the significantly enriched FA/GO terms (Bonferroni corrected log10 transformed p-values <0.05); dark grey color marks the non-significant GO terms. (B-D) Correlation analyses of *MYC* in the LCI cohort in PT, AT and HCC cell lines. (D) 28 HCC cell lines correlation profiles of 61 TER genes from the LCI cohort and of 60 random control genes. X-axis: Kendall's Tau correlation coefficient; Y-axis: cumulative relative frequency. Black circles: correlation coefficients for TER gene set (LCI); white circles: correlation coefficients for random control gene set (see Supplementary Information). Dashed lines indicate medians for correlation coefficients distributions.

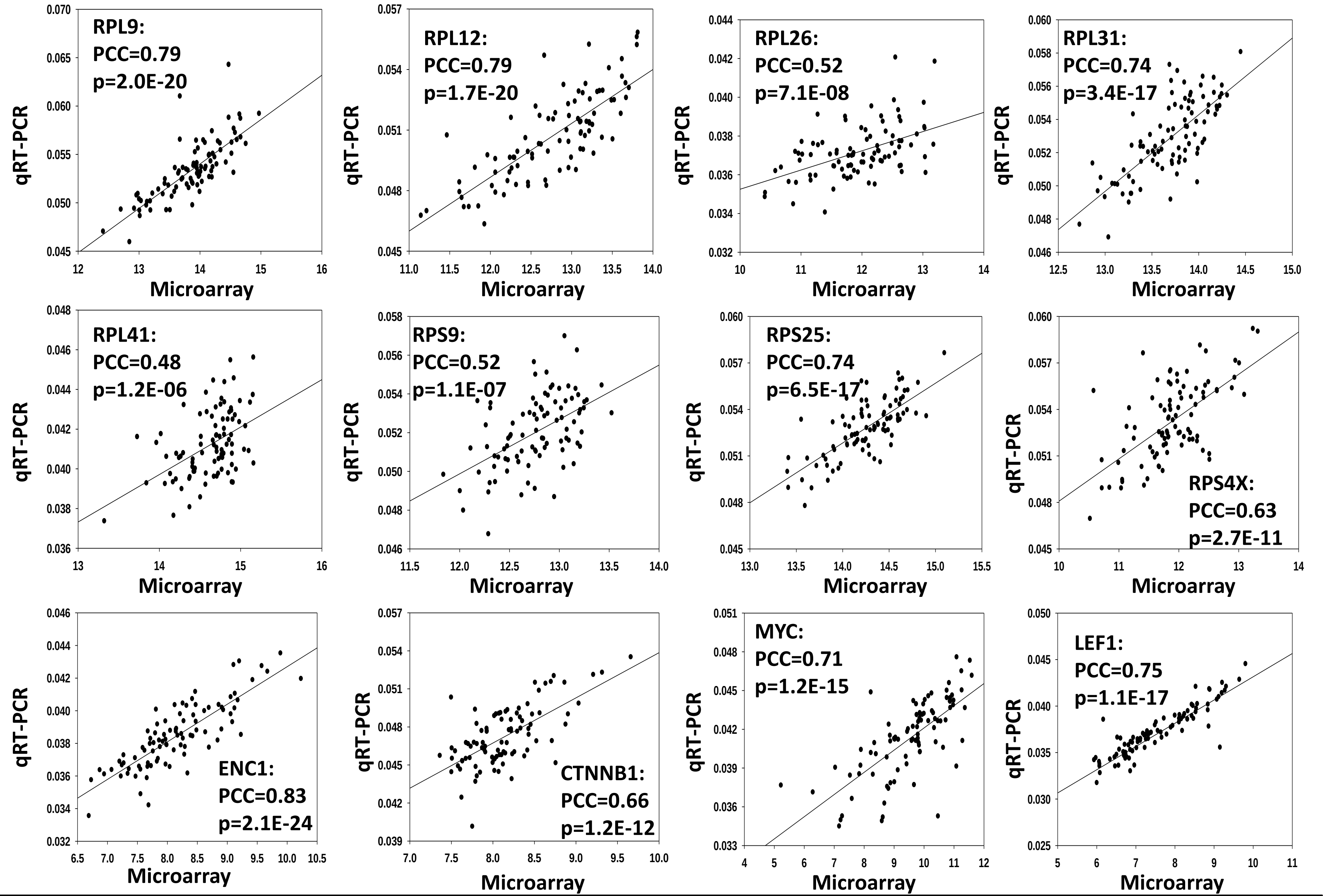

Figure S10. Concordance analysis between expression data from microarray and qRT-PCR experiments in 12 representative genes. X-axis: log2 transformed gene expression data from microarray experiments obtained for 92 randomly chosen HCC patients from the Singapore cohort; Y-axis: the qRT-PCR expression data are presented as 1/Ct values obtained after qRT-PCR experiments for the same 92 HCC patients. CC: Pearson correlation coefficient.

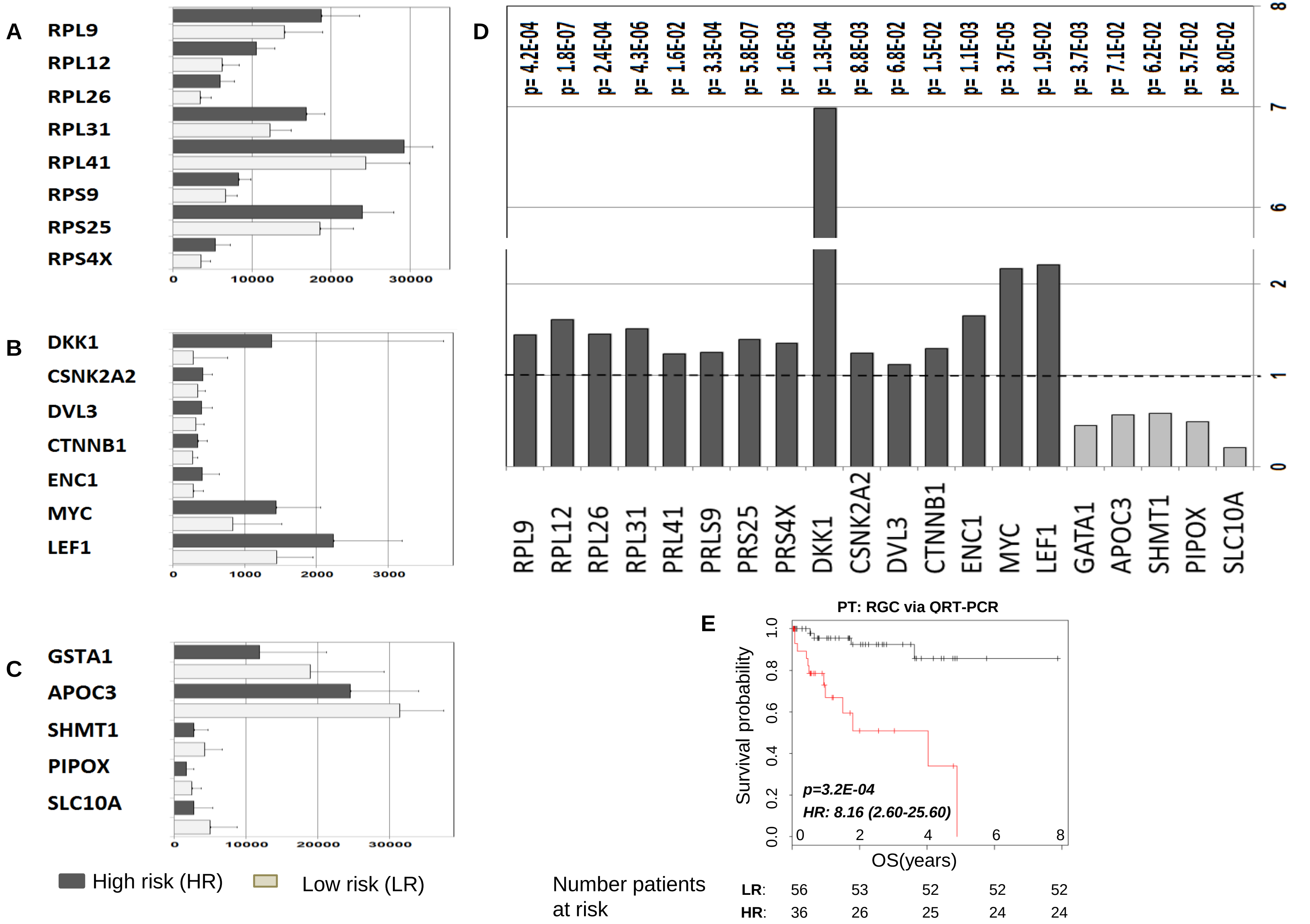

Figure S11. qRT-PCR validation of 20 representative genes either involved in the RGC or genes differentially expressed in HR_T and LR_T subgroups (see the legend in the next slide).

Figure S11. qRT-PCR validation of 20 selected genes either involved in the RGC or genes differentially expressed in HR_T and LR_T subgroups. Microarray expression in selected genes of the RGC (A), representative WNT pathway genes (B) and liver metabolism genes (C) in HR_T (dark grey bars) and LR_T(light grey bars) HCC subgroups (Singapore cohort). X-axis microarray gene expression values in linear scale. (D) qRT-PCR ΔΔCt values ($\Delta\Delta Ct_{HR,}$ or fold change) values for the same genes in HR_T subgroup. Dashed line indicates arbitrary set ΔΔCt values ($\Delta\Delta Ct_{LR}$ =1) for the every studied gene in LR_T subgroup (calibrator). In total 92 randomly selected HCC patients from the Singapore cohort were analysed. Patients stratification was according to the microarray data (Figure 2C). qRT-PCR gene expression differences (Ct values) between the HCC patients from HR_T (n=30) and LR_T (n=62) subgroups were assessed using the Mann-Whitney test (right part). Dark grey bars denote genes upregulated in HR_T subgroup patients, light grey bars: genes downregulated in HR_T subgroup. *TBP* gene was used as an internal control. (E) Kaplan-Meier survival curves for the low- and high-risk subgroups after the RGC–based stratification using qRT-PCR gene expression data in the Singapore cohort. X-axis: survival probability; Y-axis: Overall survival (years).

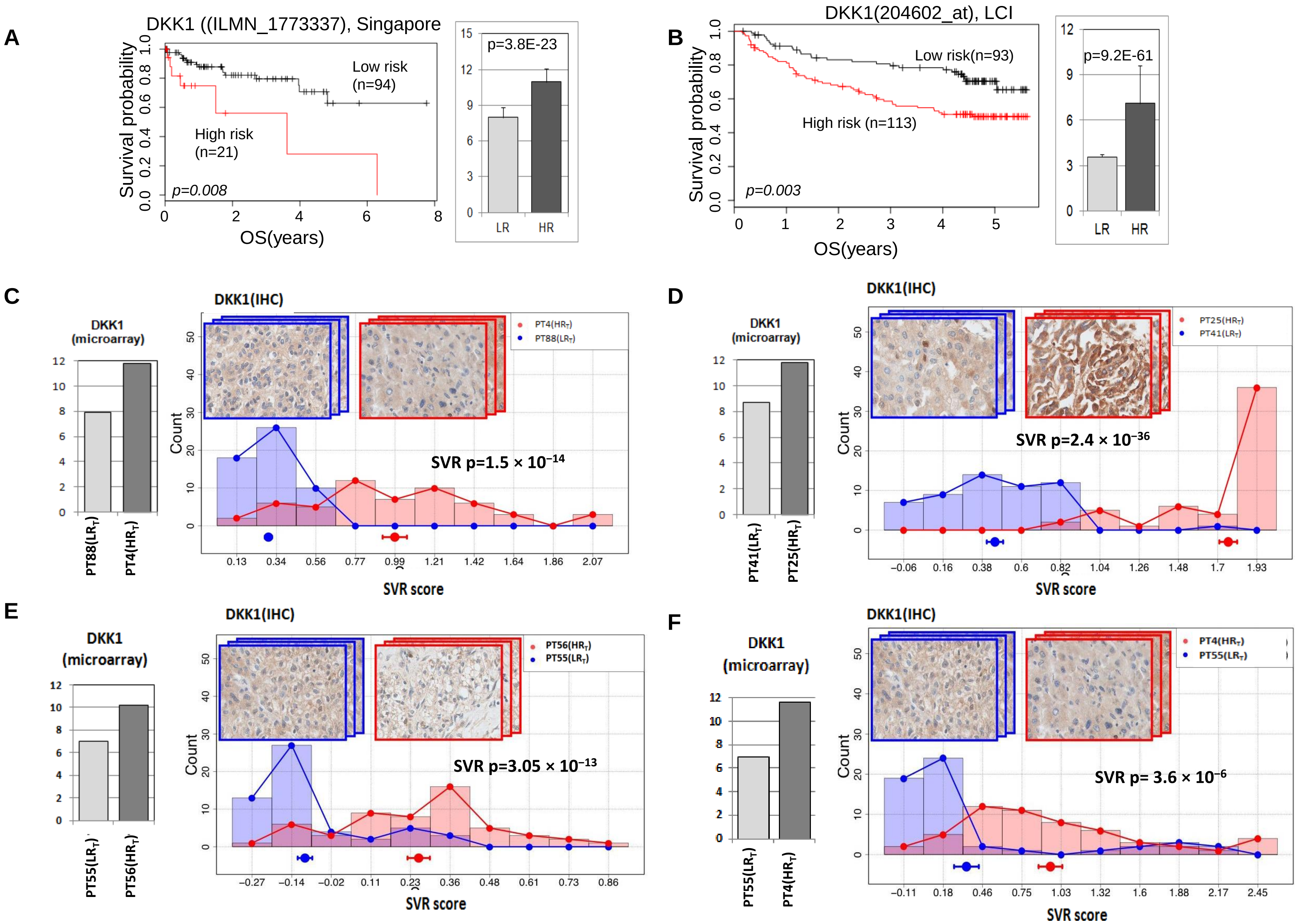

Figure S12. Results of testing of DKK1 relative expression in IHC liver tumor tissue images (PT) in 6 representative HCC patients using SVR approach (see the legend in the next slide).

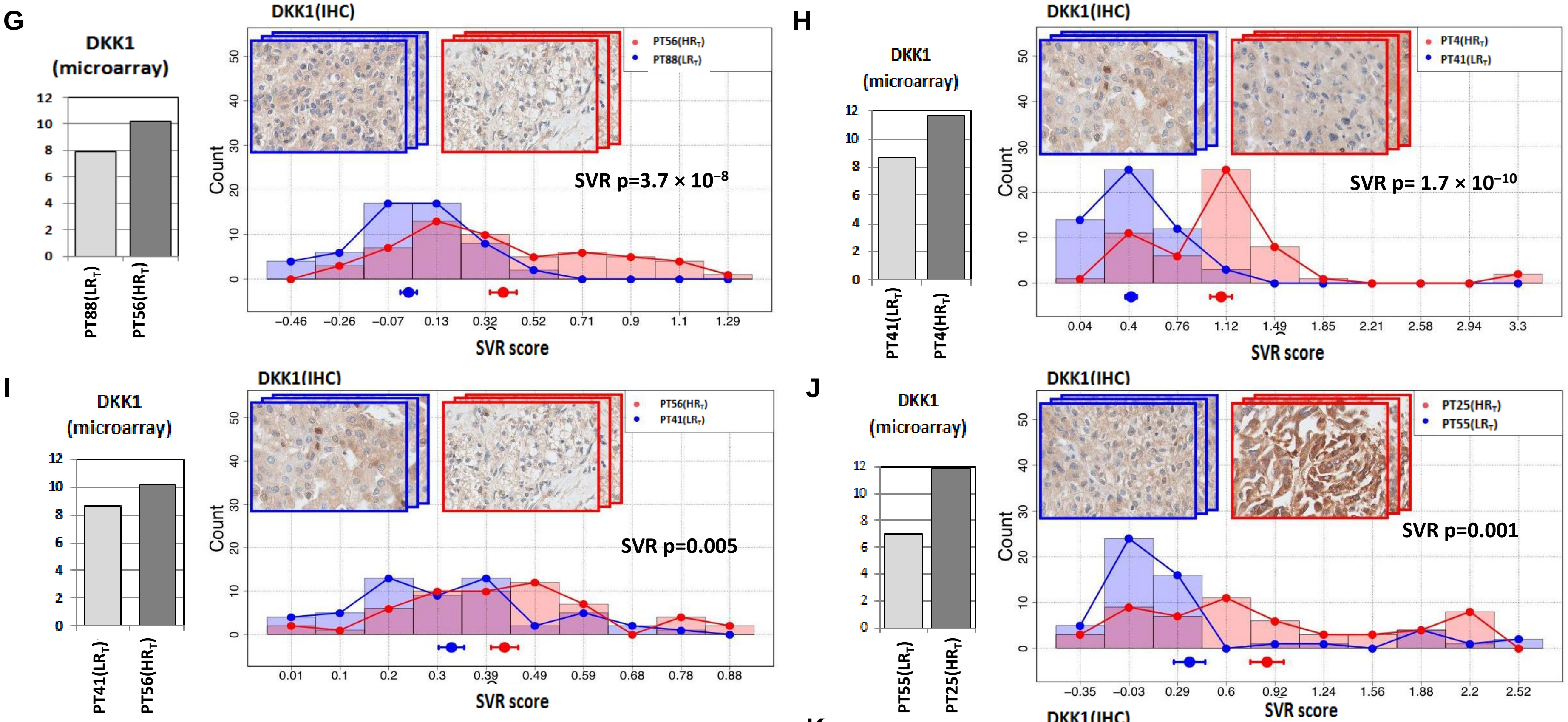

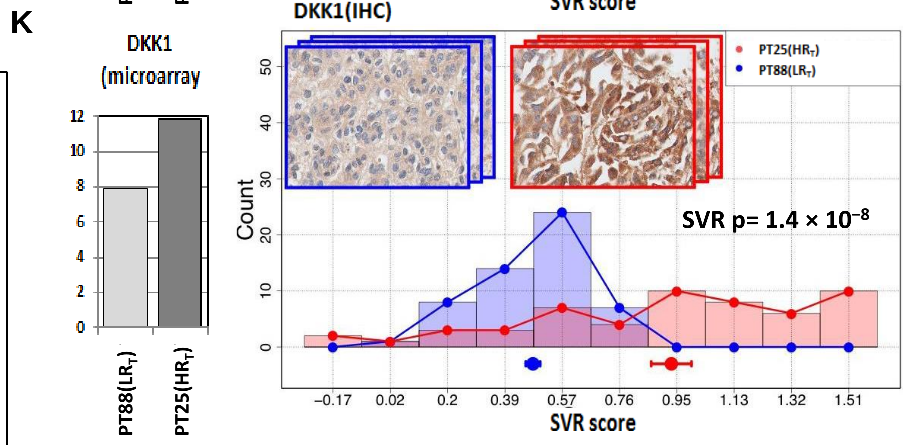

Figure S12 (continued). (A) and (B): DKK1 is pro-oncogenic on the mRNA and protein levels. Kaplan-Meier survival curves obtained by 1-D DDg using the fitting of the DKK1 microarray gene expression values to survival data in Singapore and LCI cohorts. X-axis: survival probability; Y-axis: Overall survival (years). Vertical bars and p-values show the significant difference in the level of log-2- transformed gene expression values between the low- and high-risk patient subgroups (Mann-Whitney test). LR: low risk, HR: high risk. (C)-(K): Left panels: microarray gene expression values (log 2 -transformed) in PTs for the patients; right panels show the histograms and average of predicted DKK1 Support Vector Regression (SVR) scores for tumor tissue images in the same patients. Representative sub-images of the patients pairs are also shown (Red border: HR$_T$ subgroup, Blue border: LR$_T$ subgroup). (C) PT4 (HR$_T$ subgroup) and PT88 (LR$_T$ subgroup); (D) PT41 (LR$_T$ subgroup) and PT25 (HR$_T$ subgroup); (E) P56 (HR$_T$ subgroup) and PT55 (LR$_T$ subgroup); (F) PT4 (HR$_T$ subgroup) and PT55 (LR$_T$ subgroup); (G) PT56 (HR$_T$ subgroup) and PT88 (LR$_T$ subgroup); (H) PT4 (HR$_T$ subgroup) and PT41 (LR$_T$ subgroup); (I) PT56 (HR$_T$ subgroup) and PT41 (LR$_T$ subgroup); (J) PT25 (HR$_T$ subgroup) and PT55 (LR$_T$ subgroup); (G) PT25 (HR$_T$ subgroup) and PT88 (LR$_T$ subgroup).

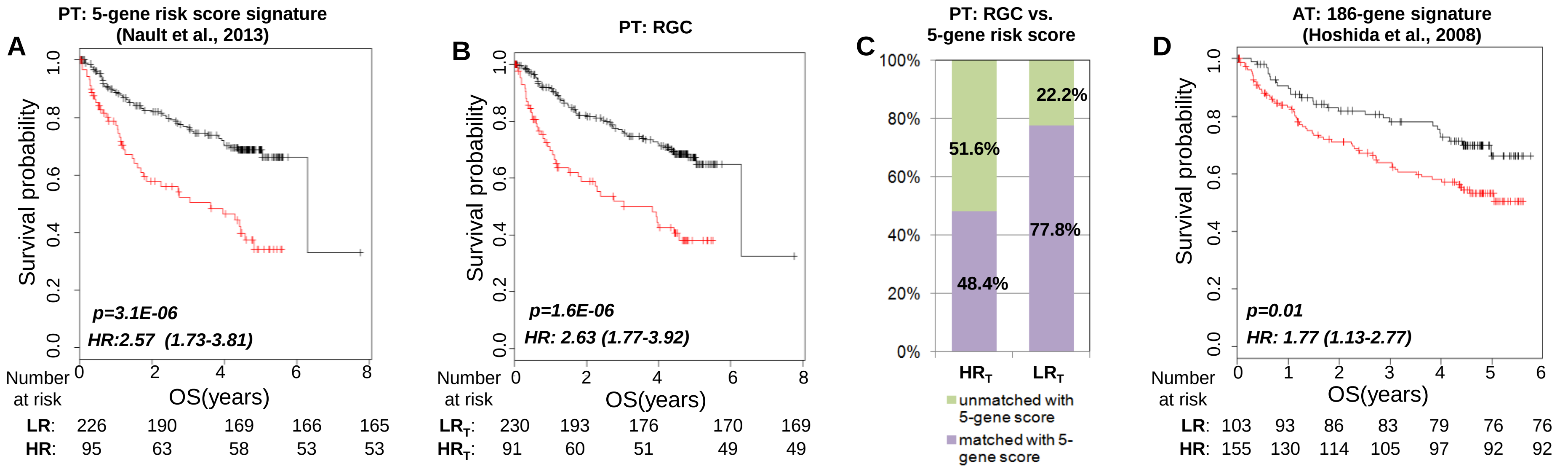

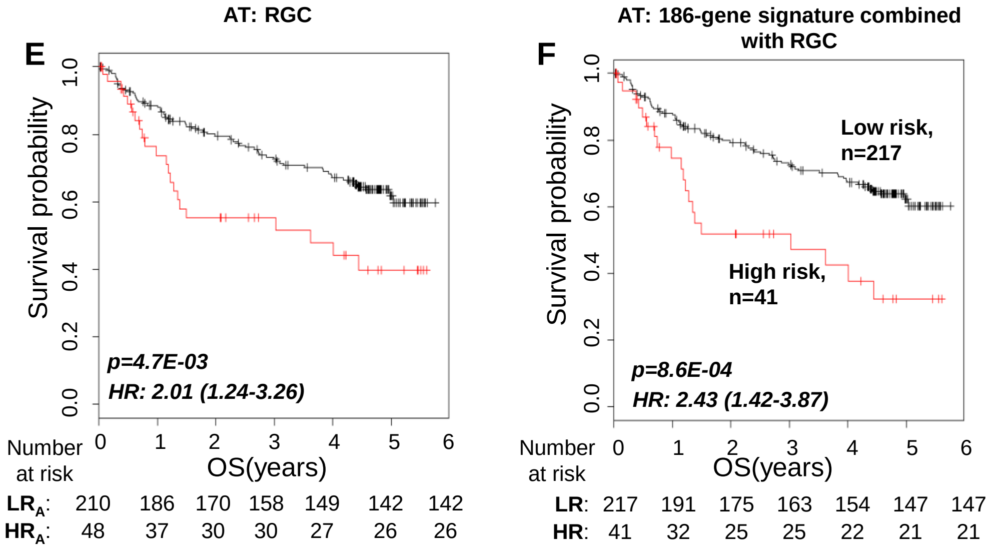

G

Multivariate analysis (PT, overall, n=321)

	HR	(CI)	Wald p-value
RGC	2.15	(1.42 - 3.25)	3.2E-04
5-gene risk score sign.	2.07	(1.37 - 3.14)	5.7E-04

Multivariate analysis (AT, overall, n=258)

	HR	(CI)	Wald p-value
RGC	1.80	(1.10 – 2.95)	0.02
186-gene survival sign.	1.62	(1.02 – 2.56)	0.04

Figure S13. Comparison of RGC prognostic power with other prognostic gene signatures. Kaplan-Meier survival curves for the low- and high-risk subgroups in the combined overall group (Singapore + LCI cohorts). X-axis: survival probability; Y-axis: Overall survival (years). LR: low risk, HR: high risk. (A) and (B): 5-gene risk score signature and RGC in PT, respectively. (C) Overlaps of patient subgroups stratified by the RGC and 5-gene score signature in PT. (D), (E) and (F) : 186-gene survival signature, RGC and their combination, respectively. The high risk subgroup in (F) corresponds to the patients who was classified into the high risks subgroups by both the RGC and 186-gene signature. (G) multivariate analysis for independent performance of the RGC adjusted to the 5-gene risk score signature (top, PT) or to the 186-gene survival signature(bottom, AT).
