# Supplementary material for: Tumor‐adjacent tissue co‐expression profile analysis reveals pro‐oncogenic ribosomal gene signature for prognosis of resectable hepatocellular carcinoma
Source: Mol Oncol. 2017 Dec 12;12(1):89–113. doi: 10.1002/1878-0261.12153 (PMC5748488; doi:10.1002/1878-0261.12153)
Supplement: Supplementary file 3 [file MOL2-12-89-s003.pdf]

Tumor-adjacent tissue co-expression profiles analysis reveals pro-oncogenic ribosomal gene signature for prognosis of resectable hepatocellular carcinoma

Oleg V. Grinchuk [1*], Surya P. Yenamandra [1*], Ramakrishnan Iyer [1], Malay Singh [1,2], Hwee Kuan Lee [1,2], Kiat Hon Lim [3], Pierce Kah Hoe Chow [3,4,5,6], Vladimir A. Kuznetsov [1,6]

[1] Bioinformatics Institute, Singapore,
[2] Department of Computer Science, National University of Singapore, Singapore,
[3] Department of Surgical Oncology, National Cancer Centre, Singapore
[4] Office of Clinical Sciences, Duke-NUS Graduate Medical School, Singapore
[5] Department of HPB and Transplantation Surgery, Singapore General Hospital, Singapore

[6] Corresponding authors:
pierce.chow.k.h@singhealth.com.sg
vladimirk@bii.a-star.edu.sg

* equal contribution

SUPPORTING INFORMATION

1. Image based analysis of immunohistochemistry slides

These tissue slides have been immunohistochemically stained using Hematoxylin and Diaminobenzidine based protocol and anti-DKK antibody. The presence of biomarker DKK1 protein in the cell was indicated by brown color in these immunohistochemistry (IHC) stained images.

HCC patients from the Singapore cohort were stratified into two HCC subgroups, namely High Risk Tumor Samples (HR_T) and Low Risk Tumor Samples (LR_T) by the tissue microarray based 24 Ribosome Gene classification system (RGC). Three representative HCC patients in each subgroup have been selected. Correspondingly, six liver tumor tissue slides were then annotated for tumor tissue regions by a trained pathologist. For each slide, six images were extracted from the annotated tumor regions.

The brown and blue colors indicate the signal for DKK1 protein and nuclei respectively in the IHC images. These images were processed by ImageJ Immunohistochemistry Analysis Toolbox for separation of brown and blue colors [1,2,3]. Only the separated “brown” images were analyzed further.

The 3-channel RGB “brown” images were converted to grayscale and thresholded using Otsu method to remove the faint brown regions for subsequent analysis [4]. The dataset of six images per patient was extended by dividing each of these images into 9 sub-images by an equally spaced 3 by 3 grid. This division was done such that the sub-images can still be visually (qualitatively) differentiated between HR_T

and LR_T subgroups and it was a good amount of pixel based information for subsequent feature extraction. If we divided more then it became harder to visually differentiate the sub-images. Also, lesser number of sub-images did not extend the dataset sufficiently.

For each sub-image, all the 3-channel “brown” color intensities were processed for calculation of Red, Blue, and Green (RGB) intensity covariance matrix. This symmetric 3×3 matrix has 6 unique variables which can be used as a feature vector of the corresponding sub-image. Each of the six patients has a total of $54(= 6 \times 9)$ sub-images in the dataset. All the sub-images from HR_T patients and LR_T patients were labeled as “1” and “0” respectively. This labeled dataset with RGB covariance matrix based features is used for training and testing of Support Vector Regression system [5]. The image processing pipeline, RGB covariance based feature extraction system, and Support Vector Regression system was implemented using ImageJ, OpenCV, C++, and R.

All the nine pairs of the three HR_T and three LR_T patients were used for testing the Support Vector Regression system after training on the other four patients. In each of these cross-validation runs there were $216(= 54 \times 4)$ sub-images for training and $108(= 54 \times 2)$ sub-images for testing.

A trained Support Vector Regression system is expected to predict a lower score (value) for LR_T patient’s sub-images when compared to HR_T patient’s sub-images. Histograms of predicted scores (by Support Vector Regression) for 6 test patients (108 sub-images) are shown in Fig. S12. In all the nine possible patients pairs the average predicted Support Vector Regression score for LR_T patient’s sub-images was always less than that of HR_T patient’s sub-images which is as per expectations. Representative sub-images of the patients have also been illustrated in these figures.

2. ChIP-seq analysis

We carried out the ChIP-seq binding regions identification in the proximal promoters of the RGC and other gene sets. CBR Data were generated by ENCODE project and visualized in UCSC genome browser (www.genome.ucsc.edu/ENCODE/). According to ENCODE, the ChIP-seq peaks were called based on the sequence clusters processed using F-Seq, a software developed by Boyle et al [6]. For each studied gene set, we localized higher confidence ChIP-seq binding regions via mapping of significant MYC ChIP-seq DNA fragments cluster overlap peaks (at p-value <0.0001). Genomic region manipulations (e.g., merging, joining, intersections of genomic region intervals) were performed using the Galaxy platform [7].

3. Comparison of standard clinicopathological parameters between the Singapore and LCI cohorts (Table 1).

Percentage of patients with HBV infection and cirrhosis significantly predominated in the LCI cohort compared to the Singapore cohort (91% vs. 46% and 92% vs. 54%, respectively). However, the percentage of patients with large tumors size (>5cm) and B stage (BCLC classification) was significantly higher in the Singapore cohort (34% vs. 57% and 9% vs. 23%, respectively). The two cohorts also displayed differences in median follow-up (1.17 years vs. 4.36 years in Singapore and LCI cohorts, respectively), median age (64 vs. 51 years, respectively) as well as in percentage of cases with overall death (22% vs. 39%) and with early recurrence (38 vs. 92%). No significant differences were observed in patients with high serum AFP level (>300 ng//mL), multinodular tumors or TNM staging. Such differences between the cohorts could be explained, most probably, by a non-identical study design between the LCI [8] and/or Singapore cohorts and/or varied accepted HCC patients diagnosis and treatment guidelines between China and Singapore [9].

4. Comparison of 1D DDg results obtained in Singapore and LGI datasets

In the contexts of robustness and reproducibility of the RGC predictors, we carried out a comparison of the 1D DDg results obtained in Singapore and LGI datasets.
Using 52 PT samples available in the paired PT-AT samples in the Singapore cohort, we identified the 1D DDg stratification characteristics using the 24 genes included in our RGC (Table S5B). Table S5A and Table S5B show a remarkable similarity of characteristic's values and the correlations of these characteristics, suggesting a robustness of the PT RGC (Table S5A). Also, we used the 1D DDg-defined stratification cut-off values of the genes, found in training dataset (115 PT samples in Singapore cohort), and carried out the LGI patient's stratification using the RGC genes detected in the LGI PT samples. At these circumstances, we observed only the pro-oncogenic expression patterns among RGC genes, and a high enrichment of the prognostic genes (14/24; Wald $P<0.1$), detected in the LGI PT samples were also the prognosticaly significant (Table S5A; Table S5C). These findings suggest a high probability of occurrence of the CPG in PT of the LGI cohort and ability to use the Singapore PT RGC characteristics as a training set.

We noticed that in total, the 1D DDg-defined prognostic characteristics, reported in Singapore and in LGI cohorts (Tables S3-S5) suggest robustness, concordance and cooperation of the TER genes expressed in the paired PT and AT samples.

5. MYC as a key regulator of ribosomal pathway in HCC PT and AT

Investigation of large enough numbers of genes with expression patterns similar to *MYC* could help to get an indication which downstream biological pathways could be predominantly regulated by *MYC* directly or indirectly. Hence, we performed the correlation analysis of the transcript encoding the *MYC* gene using the "GeneNeighbors" module from the GenePattern portal for identification of genes which most closely resemble a continuous profile for a gene of interest [10]. It was estimated that the number of functional *MYC* transcriptional targets may reach 10-15% of all protein encoding genes in the genome[11]. In each cohort both for PT and AT samples, we selected the top 2000 protein encoding genes (≈10% of the human genome) most resembling correlation profile of *MYC* (representative Illumina probe "ILMN_1680618" in the Singapore cohort and the representative Affymetrix probe set "202431_s_at" in the LCI cohort). Because TF *ELK1* was reported to regulate up to 30% of all ribosomal pathway genes in a cancer HeLa cell line[12] was studied as an alternative potential regulator of ribosomal genes in PT and AT (the Illumina probe ILMN_1654289 for the Singapore cohort and the Affymetrix probeset 210376_x_at in the LCI cohort).

Heatmap for FA/GO enrichment analysis of selected gene sets is shown in Fig.S9A. Briefly, FA/GO enrichment analysis of the top best correlated genes with *MYC* in PT and AT revealed multiple significantly enriched FA/GO terms. REVIGO [13] was used to remove redundancy of the FA/GO terms.

Importantly, that several of them were common in both tissue types in both cohorts, e.g."GO:0003723~RNA binding"(P=0.001 in PT and $P=1.5 \times 10^{-13}$ in AT in the Singapore cohort, $P=3.3 \times 10^{-41}$ in PT and $P=5.9 \times 10^{-8}$ in AT in the LCI cohort), "GO:0006414~ translation elongation" ($P=9.4 \times 10^{-17}$ in PT and $P=8.6 \times 10^{-26}$ in AT in the Singapore cohort, $P=2.0 \times 10^{-49}$ in PT and $P=2.3 \times 10^{-05}$ in AT in the LCI cohort), "structural constituent of ribosome" ($P=3.1 \times 10^{-16}$ in PT and $P=1.5 \times 10^{-26}$ in AT in the Singapore cohort, $P=2.3 \times 10^{-34}$ in PT and $P=5.1 \times 10^{-4}$ in AT in the LCI cohort). Interestingly, we also observed enrichment of such FA/GO terms as "GO:0005739~mitochondrion" ($P=2.6 \times 10^{-10}$ in PT and $P=2.3 \times 10^{-15}$ in AT in the Singapore cohort, P=0.008 in PT in the LCI cohort) and "GO:0006955~immune response"($P=1.8 \times 10^{-4}$ in PT and P=0.001 in AT in the LCI cohort). Beside the ribosomal pathway, *MYC* was reportedly shown to be a regulator of several other pathways in cancers including genes of tumor immune microenvironment[14,15] and mitochondrial biogenesis [16]. Because gene sets involved in translation elongation and ribosomes showed the strongest FA/GO enrichments in PT and AT in both cohorts, we suggested them as possible downstream targets of *MYC* in the studied HCC cohorts. In contrast, the top positively correlated genes with *ELK1* yielded no enriched FA/GO terms related to translation or ribosomes; therefore, *ELK1* might be not the major regulator of the ribosomal pathway in the studied HCC cohorts.

In the next set of experiments (Fig. 5A, 5B and Fig. S9B - S9D) we assumed that the degree of correlation of *MYC* expression with expression values of a given gene set may reflect a degree of a regulatory effect of *MYC* on gene expression in the same gene set. The larger number of genes positively correlated with *MYC* in the set would imply the stronger regulatory effect of *MYC* in the set. Therefore, here we compared a potential regulatory effect of *MYC* on the TER gene sets (Tables S3 and S4) in two studied HCC cohorts and the set of 28 HCC cell lines. As a negative control gene set, we used the 60 protein encoding genes randomly chosen from the genome (Table S10).

We compared the cumulative frequency distribution functions of the Kendal's Tau correlation coefficient values estimated between gene expression values of *MYC* and the TER gene sets and between the *MYC* and the negative control gene set (Fig. 9B - 9D). TER gene sets included the genes overrepresented under the GO term " GO:0006414~translation elongation" identified either in the Singapore cohort (44 TER genes (Singapore), Fig. 1F, Table S3) or in the LCI cohort (61 TE genes(LCI)) (Fig. S5A, Table S4). Significant overall positive shifts (by Kolmogorov-Smirnov test) of correlation coefficient values between the TE gene sets and *MYC* expression within the same tissue (either PT or AT) was observed. Specifically, the significance of the TE gene sets and the negative control gene set were characterized by $P=8.4 \times 10^{-8}$ in PT and $P=4.2 \times 10^{-12}$ in AT from the Singapore cohort and by $P=3.2 \times 10^{-17}$ in PT and $P=0.003$ in AT from the LCI cohort. The 28 HCC cell lines (transcriptome expression data (Affymetrix U133Plus platform) were obtained from the Cancer Cell Line encyclopedia at Broad Institute: http://www.broadinstitute.org/ccle/home) displayed also the positive shift (at $P=3.4 \times 10^{-4}$).

On the next step, we studied direct *bona fide MYC* transcription targets using the ChIP-Seq TF binding data for the HepG2 HCC cell line, which is one of the representative 28 HCC cell lines analyzed in Fig. S9D. The vast majority of ribosomal genes in two TER gene sets and all genes of the RGC revealed strong signals for *MYC* ChIP-seq binding regions in proximity of their promoters (Table S10). Fig.5C demonstrates the strong enrichment of unique *MYC* ChIP-seq binding regions overlaps with proximal promoters of the TER gene sets (derived from the Singapore and the LCI cohorts) as well as for all 24 genes of the RGC (Fisher's exact test) as compared to the random genes control ($P=1.5 \times 10^{-13}$, $P=2.2 \times 10^{-20}$ and $P=5.5 \times 10^{-12}$, respectively). Noteworthy, the difference in frequencies of ChIP-seq binding regions overlaps between the random gene control set and total genome gene set (23930 unique Gene Symbols IDs: hg19 RefSeq track of the UCSC Genome Browser) was insignificant ($P=0.3$). *MYC* ChIP-seq binding regions were enriched in differentially upregulated gene set in HR_T subgroup ($P=1.3 \times 10^{-11}$). The finding that all genes of the RGC can be driven by the same regulator *MYC* highlights the power of

our original approach for selection of mechanistically- and uniformly-driven prognostic biomarkers of HCC. Additionally, proximal promoter analysis of DEGs in PT obtained after RGC stratification (see Results) indicates that most of the upregulated DEGs (66.7%) in the HR_T subgroup might be driven by *MYC* (Fig.5D).

6. RGC-based HCC stratification performance

We compared the prognostic performance of the RGC in PT to the 65-gene risk signature [17], 16-gene G1-G6 signature[18], vascular invasion gene signature [19] and the 5-gene score signature by Nault et al [20]. Additionally, the prognostic performance of the RGC in AT was compared to the 186-gene survival signature (Hoshida et al.[21]), because it was suggested previously as a promising prognostic biomarker in AT. Firstly, we used the 1-D DDg procedure to select and compare the most optimal patient's partition into low- and high-risk subgroups for every gene with the best Wald *P*-values (the training mode). Next, we applied exactly the same computational procedures (1-D DDg and SWVg) which were used for the training and cross-cohort validation of the RGC [22,23,24] to all five gene signatures to ensure their unbiased comparison. We fixed the most optimal parameters (gene expression value cutoff, partition design and SWVg score) for the genes in four gene signatures in the Singapore cohort and generated prognosis prediction blind to survival data in the LCI cohort.

7. Potential therapeutic intervention strategies after RGC-based HCC risk stratification

Potential therapeutic intervention strategies after RGC stratification may include:

i) the already existing and proven to be efficient in HCC treatment with sorafenib, which can also significantly modulate the deregulated *WNT* signalling [25];

ii) key members of the *WNT* pathway (*DKK1*, *LEF1, CTNNB1* and *MYC*) (e.g., anti-DKK1 antibody drug BHQ880A was involved in Phase II clinical trial in high risk multiple myeloma patients by Novartis Pharmaceuticals (Trial ID: NCT01302886) [26];

iii) *TGFBR2*, as a potential drug target common for HCC PT and AT (current report), is involved in phase I clinical trial in solid tumors by Eli Lilly (Trial ID:NCT01646203) and had been recently proposed as a promising drug target for HCC) [27,28];

iv) focal adhesion pathway known as an important factor in radio- and chemoresistance in many cancers[29];

v) micro-vesicles/exosomes as potential biologic messengers of tumor growth signals[30];

vi) genes encoding ribosomal proteins and their products [31,32];

vii) common and unique targets in DNA repair and damage regulatory pathways in PT and AT

viii) tumor supressors negatively correlated with RGC and TER genes in PT and AT (e.g., FBP1 and SPOP)

Each of the potential intervention targets and their combinations may be directly tested using experimental biological models with potential translation in clinical trials of HCC in the high-risk prognostic subgroups after the RGC-based patient prognosis. Among all mentioned above, specific targeting common prognostic genes and pathways in PT and AT might represent an alternative avenue in HCC (on the several other high-aggressive neoplasms) treatment and diagnostics due to the strategy considers the host and cancer tissues as a whole targeting organ with its common and unique pathological dysfunction states.

8. SUPPLEMENTARY REFERENCES

1. (2014)“Immunohistochemistry(IHC) Image Analysis Toolbox.” https://imagej.nih.gov/ij/plugins/ ihc-toolbox/index.html, 2014. [Online; Accessed 30-May-2016].
2. Shu J, Qiu G, I. M (2013) A semi-automatic image analysis tool for biomarker detection in immunohistochemistry analysis. “A semi-automatic image analysis tool for biomarker detection in immunohistochemistry analysis,” in Image and Graphics (ICIG), 2013 Seventh International Conference on, pp 937–942, IEEE, 2013.
3. Shu J, Qiu G, Ilyas M, Kaye P (2010) Biomarker detection in whole slide imaging based on statistical color models. MICCAI 2010 Workshop on Computational Imaging Biomarkers for Tumors: From Qualitative to Quantitative.
4. Otsu N (1975) A threshold selection method from gray-level histograms. Automatica 11: 23–27.
5. Smola A, Vapnik V (1997) Support vector regression machines. Advances in neural information processing systems 9: 155-161.
6. Boyle AP, Guinney J, Crawford GE, Furey TS (2008) F-Seq: a feature density estimator for high-throughput sequence tags. Bioinformatics 24: 2537-2538.
7. Goecks J, Nekrutenko A, Taylor J (2010) Galaxy: a comprehensive approach for supporting accessible, reproducible, and transparent computational research in the life sciences. Genome Biol 11: R86.
8. Roessler S, Jia HL, Budhu A, Forgues M, Ye QH, et al. (2010) A unique metastasis gene signature enables prediction of tumor relapse in early-stage hepatocellular carcinoma patients. Cancer Res 70: 10202-10212.
9. Han KH, Kudo M, Ye SL, Choi JY, Poon RT, et al. (2011) Asian consensus workshop report: expert consensus guideline for the management of intermediate and advanced hepatocellular carcinoma in Asia. Oncology 81 Suppl 1: 158-164.
10. Slonim D.K. TP, Mesirov J., Golub T.R., Lander E. (2000) Class prediction and discovery using gene expression data. Proc of the Fourth Ann Internat Conf on Comp Mol Biol (RECOMB) 2000: 263-272.
11. Zeller KI, Zhao X, Lee CW, Chiu KP, Yao F, et al. (2006) Global mapping of c-Myc binding sites and target gene networks in human B cells. Proc Natl Acad Sci U S A 103: 17834-17839.
12. Boros J, Donaldson IJ, O'Donnell A, Odrowaz ZA, Zeef L, et al. (2009) Elucidation of the ELK1 target gene network reveals a role in the coordinate regulation of core components of the gene regulation machinery. Genome Res 19: 1963-1973.

13. Supek F, Bosnjak M, Skunca N, Smuc T (2011) REVIGO summarizes and visualizes long lists of gene ontology terms. PLoS One 6: e21800.
14. Coulouarn C, Factor VM, Conner EA, Thorgeirsson SS (2011) Genomic modeling of tumor onset and progression in a mouse model of aggressive human liver cancer. Carcinogenesis 32: 1434-1440.
15. Sodir NM, Swigart LB, Karnezis AN, Hanahan D, Evan GI, et al. (2011) Endogenous Myc maintains the tumor microenvironment. Genes Dev 25: 907-916.
16. Wahlstrom T, Henriksson MA (2015) Impact of MYC in regulation of tumor cell metabolism. Biochim Biophys Acta 1849: 563-569.
17. Kim SM, Leem SH, Chu IS, Park YY, Kim SC, et al. (2012) Sixty-five gene-based risk score classifier predicts overall survival in hepatocellular carcinoma. Hepatology 55: 1443-1452.
18. Boyault S, Rickman DS, de Reynies A, Balabaud C, Rebouissou S, et al. (2007) Transcriptome classification of HCC is related to gene alterations and to new therapeutic targets. Hepatology 45: 42-52.
19. Minguez B, Hoshida Y, Villanueva A, Toffanin S, Cabellos L, et al. (2011) Gene-expression signature of vascular invasion in hepatocellular carcinoma. J Hepatol 55: 1325-1331.
20. Nault JC, De Reynies A, Villanueva A, Calderaro J, Rebouissou S, et al. (2013) A hepatocellular carcinoma 5-gene score associated with survival of patients after liver resection. Gastroenterology 145: 176-187.
21. Hoshida Y, Villanueva A, Kobayashi M, Peix J, Chiang DY, et al. (2008) Gene expression in fixed tissues and outcome in hepatocellular carcinoma. N Engl J Med 359: 1995-2004.
22. Kuznetsov VA, Senko OV, Miller LD, Ivshina AV. (2006) Statistically Weighted Voting Analysis of Microarrays for Molecular Pattern Selection and Discovery Cancer Genotypes. IJCSNS Int J of Comput Sci Netw Secur 6: 73-83.
23. Motakis E, Ivshina, A.V. & Kuznetsov, V.A. (2009) Data-driven approach to predict survival of cancer patients: estimation of microarray genes' prediction significance by Cox proportional hazard regression model. IEEE Eng Med Biol Mag 28: 58-66.
24. Tang Z, Ow GS, Thiery JP, Ivshina AV, Kuznetsov VA (2014) Meta-analysis of transcriptome reveals let-7b as an unfavorable prognostic biomarker and predicts molecular and clinical subclasses in high-grade serous ovarian carcinoma. Int J Cancer 134: 306-318.
25. Lachenmayer A, Alsinet C, Savic R, Cabellos L, Toffanin S, et al. (2012) Wnt-pathway activation in two molecular classes of hepatocellular carcinoma and experimental modulation by sorafenib. Clin Cancer Res 18: 4997-5007.
26. Pez F, Lopez A, Kim M, Wands JR, Caron de Fromentel C, et al. (2013) Wnt signaling and hepatocarcinogenesis: molecular targets for the development of innovative anticancer drugs. J Hepatol 59: 1107-1117.
27. Morris SM, Baek JY, Koszarek A, Kanngurn S, Knoblaugh SE, et al. (2012) Transforming growth factor-beta signaling promotes hepatocarcinogenesis induced by p53 loss. Hepatology 55: 121-131.
28. Neuzillet C, de Gramont A, Tijeras-Raballand A, de Mestier L, Cros J, et al. (2013) Perspectives of TGF-beta inhibition in pancreatic and hepatocellular carcinomas. Oncotarget 5: 78-94.
29. Eke I, Cordes N (2015) Focal adhesion signaling and therapy resistance in cancer. Semin Cancer Biol 31: 65-75.
30. Kosaka N, Yoshioka Y, Tominaga N, Hagiwara K, Katsuda T, et al. (2014) Dark side of the exosome: the role of the exosome in cancer metastasis and targeting the exosome as a strategy for cancer therapy. Future Oncol 10: 671-681.
31. Gupta R, Kim S, Taylor MW (2012) Suppression of ribosomal protein synthesis and protein translation factors by Peg-interferon alpha/ribavirin in HCV patients blood mononuclear cells (PBMC). J Transl Med 10: 54.
32. Li W, Zhu C, Chen X, Li Y, Gao R, et al. (2011) Pokeweed antiviral protein down-regulates Wnt/beta-catenin signalling to attenuate liver fibrogenesis in vitro and in vivo. Dig Liver Dis 43: 559-566.
